# Three phosphatidylcholines as a potential early-diagnostic signature for hyperglycemia in preschool-aged children: a cross-sectional pilot study

**DOI:** 10.3389/fendo.2026.1878086

**Published:** 2026-08-05

**Authors:** Haifeng Zhang, Jing Wang, Yueguang Lv, Yiying Huang, Yuanyuan Guo, Chang Jiang, Ting Zhang, Gongshu Liu, Fangfang Chen

**Affiliations:** 1 Beijing Municipal Key Laboratory of Child Development and Nutriomics, Capital Center for Children’s Health, Capital Medical University, Capital Institute of Pediatrics, Beijing, China; 2 Tianjin Women and Children’s Health Center, Tianjin, China; 3 Key Laboratory of Consumer Product Quality Safety Inspection and Risk Assessment, State Administration for Market Regulation, Beijing, China; 4 Department of Epidemiology, Capital Center for Children’s Health, Capital Medical University, Capital Institute of Pediatrics, Beijing, China

**Keywords:** diagnostic signature, glycerophopholipids, lipid ratio, lipidomics, pediatric hyperglycemia

## Abstract

**Objective:**

Although lipid metabolism dysregulation commonly accompanies elevated blood glucose, lipidomic studies in hyperglycemic children remain limited. We hypothesized that distinct lipid metabolic signatures and derived lipid ratios are associated with pediatric hyperglycemia and may aid in its identification.

**Methods:**

Based on the Infant Development and Health Cohort, 59 preschool-aged children (3–4 years) with fasting plasma glucose (FPG) ≥ 5.6 mmol/L were randomly selected from 210 participants, alongside 60 matched healthy controls (FPG < 5.6 mmol/L). Semiquantitative lipidomics was performed, and lipid ratios were calculated from identified characteristic lipids. Univariate and multiple regression analyses were conducted.

**Results:**

After adjusting for age, sex, body mass index (BMI), early-life risk factors, and metabolic disorder factors, six lipid species remained independently associated with FPG variations (Bonferroni-adjusted *p* < 0.05). Lipid ratios derived from these six lipids differed significantly between groups; the decreased levels of phosphatidylethanolamine/diacyl-phosphatidylcholine was significantly correlated with elevated FPG, whereas elevated levels of ether-phosphoatidylcholine/diacyl-phosphoatidylcholine ratios was significantly associated with elevated FPG. Three phosphatidylcholine species showed good diagnostic performance (AUC = 0.811, 95% CI: 0.733–0.881). Their composite signature outperformed both the baseline model (age, sex; AUC: 0.882 vs. 0.584, DeLong’s *p* < 0.001), and the extended risk factor model (age, sex, BMI, and early-life riskr factors, AUC: 0.882 vs. 0.635, DeLong’s *p* < 0.001). The robustness of the three PCs was further supported by sensitivity analyses, which demonstrated consistent performance using Firth penalized regression (AUC = 0.883) and an alternative FPG cutoff at the 75th percentile (AUC = 0.834).

**Conclusions:**

In this cross-sectional targeted lipidomics study, a three PC lipid panel was independently associated with subclinical hyperglycemia in preschool-aged children and showed robust diagnostic performance. Two lipid ratios were also associated with FPG. These findings identify a potential lipid panel for early risk detection in this understudied population, pending validation in larger cohorts.

## 1 Introduction

Diabetes is a major global public health challenge, particularly in developing countries. A recent *Lancet* report estimated that over 800 million adults live with diabetes globally, 148 million of whom are Chinese (1). The IDF’s 10th Diabetes Atlas documented 1.2 million cases of type 1 diabetes in children under 20 years wordwide (2). Hyperglycemia, defined as fasting plasma glucose (FPG) ≥ 5.6 mmol/L according to the American Diabetes Association (ADA) (3), is a well-established risk factor for progression to type 2 diabetes (4). Even mild glucose elevtions below the diabetic threshold are associated with increased diabetes-related morbidity and mortality (5). Epidemiological evidence show 8%–24% of hyperglycemic adults develop type 2 diabetes (6), with risk rising once FPG levels exceeds 4.4 mmol/L (80 mg/dL) (7). While hyperglycemia is less prevalent in preschool-aged children (3–4 years old) than in older children and adolescents, its presence in early childhood signals underlying metabolic dysregulation that warrants close attention. The globalprevalence of impaired fastig glucose (IFG) in children aged 0–9 year is estimated at 2.51% (95% CI, 1.60%–3.41%) (8), whereas a study in Tianjin, China, reported 1.18% among children aged 5–6 years (9). Over the recent decades, diabetes and prediabetes rates among children and adolescents have risen sharply worldwide (10–12).

Lipid metabolism is dynamically regulated in early life and differs markedly from that in adults. Rapid physical growth demands extensive lipids for energy supply and cell membrane construction, leading faster metabolic turnover rate (13, 14). Concurrently, breastfeeding, complementary feeding, and early nutritional environment may exert programming effects on lipid metabolism that become manifest during this developmental period (15–17). Recent advances in lipidomics have enable the exploration of early metabolic biomarkers of hyperglycemia. In adult and adolescent populations, specific lipid species, including phsophatidylcholines (PC), and sphingomyelins (SM), have been demonstrated to correlate closely with glucose homeostasis and insulin resistance (18–22), suggesting lipid disturbances in the pathophysiology of hyperglycemia.

However, critical knowledge gaps remain regarding the preschool-age population. Unlike adults, in whom metabolic architecture has been largely consolidated and exhibits significantly reduced plasticity, as evidenced by a recent study showing that even healthy older adults display suppressed glucose kinetics and sustained gluconeogenesis following a glucose challenge, compared with younger adults, despite comparable respiratory exchange ratios (23), preschool-aged children are at a fundamentally different developmental stage. This qualitative difference renders extrapolation from adult or even older pediatric cohorts scientifically unjustifiable. Several critical knowledge gaps arise from this unmet need. First, existing pediatric lipidomic studies have almost exclusively enrolled school-aged children or adolescents (typically >7 years), and most have adopted case-control designs comparing clinically diagnosed patients with healthy controls. Preschool-aged children, especially those aged 3–4 years, remain largely unstudied, despite being situated at a critical period for metabolic trajectory formation (24). Second, subclinical metabolic alterations in preschool-aged children with FPG levels above the optimal range but below diagnostic thresholds (i.e., the prediabetes) have not been elucidated in this age group. Third, pragmatic lipidomic biomarker screening tools for this asymptomatic population are currently lacking. To our knowledge, no study has systematiclly investigated the association between comprehensive plasma lipidomic profiles and hyperglycemia in children, a gap that limits our understanding of the metabolic perturbations triggered by hyperglycemia during this developmental stage and their underlying pathophysiological mechanisms.

Lipidomics has become a vital tool for early detection and mechanistic investigation of diabetes, as it can identify subtle metabolic disturbance preceding changes in conventional clinical biomarkers. Studies in adults have demonstrated that lipid-related biomarkers effectively predict prediabetes and type 2 diabetes. Combined panels containing lysophosphatidylcholine (LPC), PC, phosphoatidylethanolamine (PE), ceramide-to-SM ratios and triglycerides (TAGs) achieve high diagnostic efficiency for prediabetes and type 2 diabetes (25). Altered proportions of glycerophospholipids, sphingolipids and fatty-acid components are closely linked to prediabetic status (26). Moreover, the balance between PC and PE has important implications for insulin sensitivity: human skeletal-muscle studies revealed insulin sensitivity is modulated by PC and PE contents as well as PC/PE ratios (27), and disruption of PC-PE homeostasis in liver and muscle can trigger insulin resistance, obesity and fatty-liver disease (28, 29). In pediatric cohorts, findings from the Finnish DIPP cohort demonstrated reduced SM levels occur before islet autoimmunity and subsequent type 1 diabetes onset among infants and young children (30, 31). However, these pediatric lipidomic investigations have primarily focused on type 1 diabetes-associated autoimmunity, leaving hyperglycemia-related lipid remodeling largely unexplored in preschool-aged children.

The health consequences of childhood hyperglycemia underscore the urgency of this study. Without timely intervention, childhood hyperglycemia can trigger acute complications such as ketoacidosis, cardiovascular and neurological damage, stunted growth, and increase long-term diabetes risk. Nevertheless, existing pediatric studies have predominantly focused on neonatal hyperglycemia in preterm infants, which adversely affects neuronal and retinal development and correlates with poor outcomes (32); or on disease-related hyperglycemia in hospitalized children, linked to adverse clinical events (33). Emerging evidence further suggests acute hyperglycemia impairs children’s emotional and behavioral functions over cognitive or motor abilities (34), whereas community-dwelling preschoo-aged children with asymptomatic glycemic elevation remain virtually uninvestigated.

In view of these gaps, the present study aims to systematically characterize the plasma lipidomic profiles associated with hyperglycemic status in preschool-aged children. The specific objectives are: (1) to identify plasma lipid species that are significantly associated with FPG and to examine the correlations between selected core lipid class ratios and glycemic levels; (2) to screen candidate lipid biomarkers capable of effectively discriminating hyperglycemic status; and (3) evaluate whether the addition of lipid biomarkers to traditional risk factors significantly improves the discriminative ability for hyperglycemic status in preschool-aged children. Given the paucity of lipidomic data in children at this stage, our study provides new pathophysiological insights into lipid metabolism dysregulation associated with subclinical hyperglycemia in this understudied population, and identifies a candidate lipid panel that may facilitate early risk stratification.

## 2 Materials and methods

### 2.1 Study design

This study represents a cross-sectional analysis of data derived from the ambidirectional Infant Development and Health Cohort (2017–2020) (35–37).

### 2.2 Study population

Preschool-aged children (3–4 years old) were recruited using cluster sampling from 42 public kindergartens in Tianjin, China, from September 2017 to October 2018. This age group was selected because the screening protocol could be feasibly integrated into routine kindergarten health chack-up and fasting venous blood collection was operationally achievable with standardized procedures. Among the initial cohort of 3344 children screened, 210 children (134 boys and 76 girls) met the criteria for hyperglycemia criterion (FPG ≥ 5.6 mmol/L). From these, 30 boys and 30 girls were randomly selected as the hyperglycemia group (HG; with one sample excluded due to hemolysis), and 60 age- and sex-matched normoglycemic children (FPG < 5.6 mmol/L, 30 boys and 30 girls) were randomly selected as controls (Ctr).

Exclusion criteria were: (1) inability to provide informed consent; (2) presence of any condition, chronic disease, or use of medication known to affect growth and development; (3) acute illnesses preventing physical examination; (4) multiple births; and (5) inability to collect blood samples.

Inclusion criteria for the HG group were: (1) FPG ≥ 5.6 mmol/L; (2) absence of elevated TG, total cholesterol (TC), low-density lipoprotein–cholesterol (LDL-C), high-density lipoprotein–cholesterol (HDL-C), or uric acid; (3) no overweight or obesity; and (4) absence of other diseases influencing glucose metabolism.

Inclusion criteria for the Ctr group were: FPG < 5.6 mmol/L; normal lipids and uric acid levels; and absence of overweight, obesity, or other acute or chronic diseases.

### 2.3 Data collection and measurements

Early-life data (family income, maternal age (AgeMum), pre-pregnancy BMI (mBMI), gestational weight gain (GWG), ponderal index (PI), gestational age, breastfeeding status within the first 6 months (breastfeeding), and the 0–2 year growth trajectory) were extracted from maternal and child health (MCH) records, which included routine medical and anthropometric data from pregnancy to age 2 years (37). These early-life factors were selected based on their established associations with childhood adiposity and metabolic health (38).

Anthropometric measurements were conducted as described previously (20). Height was measured without shoes. Body composition, including weight, fat mass, and percentage of body fat mass (FM%), was assessed using a body composition analyzer (Seehigher BAS-H, Beijing, China) with bioelectrical impedance analysis (BIA) by trained nutritionists. BIA was chosen for its non-invasive nature, ease of administration, and feasibility for large-scale community-based screening in young children (39). Body mass index (BMI) was calculated as weight (kg) divided by height squared (m²). BMI z-scores (zBMIs) were derived based on World Health Organization measures standards (40).

To capture heterogeneous growth patterns that better predict later metabolic risk than single-point measures (41), a latent class growth model (LCGM) was applied to model zBMI trajectories from birth to 24 months. The children were classified into three trajectories, namely, low, middle, and high (17). Model selection was based on higher entropy, lower akaike Information Criterion (AIC), bayesian Information Criterion (BIC), and adjusted BIC (aBIC), along with significant Lo-Mendell-Rubin (LMR) and bootstrapped Likelihood Ratio Test (BLRT) *p*-values (**Supplementary Table 1**). The LCGM identified three trajectory classes: a low-trajectory group (zBMI between -0.5 and 0; n = 873, 27.14%), a middle-trajectory group (zBMI between 0 and 0.5; n = 1762, 54.77%), and a high-trajectory group (characterized by a steep rise from birth, peaking at 6–9 months, followed by a gradual decline but remaining elevated compared with other groups; n = 582, 18.09%).

Fasting venous blood samples were collected at the age3 years For biochemical assays of FPG, TG, TC, LDL-C, and HDL-C, using a Hitachi 7060C Automatic Biochemistry Analysis System (Hitachi, Tokyo, Japan) following standard protocols (42).

### 2.4 Lipidomic analysis

Fasting plasma samples were collected in ethylenediaminetetraacetic acid tubes, immediately processed, and stored at −80 °C until analysis. Targeted lipidomic profiling was performed using a semiquantitative approach as previously described (43), which provides detailed LC-MS/MS parameters, including chromatographic conditions, gradient program, and MS source settings. In brief, 95 μL plasma was spiked with 5 μL of internal standard mixture (Avanti Splash Lipidomix™; Avanti Polar Lipids, Inc., Alabaster, AL, USA), and lipids were extracted via cold isopropanol protein precipitation. Extracts were analyzed by ultrahigh-performance liquid chromatography–tandem mass spectrometry (Waters, Milford, MA, USA) equipped with an electrospray ionization (ESI) source. Separation was performed on a Waters Acquity UPLC BEH Amide column (1.7 μm, 2.1 × 100 mm) using mobile phases of (A) 95% acetonitrile/10 mmol/L ammonium acetate and (B) 50% acetonitrile/10 mmol/L ammonium acetate. Detection was conducted in MRM mode using MassLynx v4.1. Lipid identification was confirmed by retention time matching against an in-house library and by the co-elution of two MRM transitions for each lipid species, with identification confidence set at Level 2 according to the LSI criteria. Quality control (QC) samples were injected after every 11 experimental runs to ensure analytical reliability. Analytical reproducibility was assessed by calculating the coefficient of variation (CVs) of lipid species in pooled QC samples. Lipid species with CVs ≤ 30% in QC samples were retained for further analysis, indicating acceptable method precision for the majority of detected lipids.

### 2.5 Statistical analysis

#### 2.5.1 Descriptive and univariate analyses

Continuous and categorical variables were analyzed using distinct software tools. Continuous data were analyzed with the compareGroups package in R and are reported as means ± standard deviations and number (mean ± SD, n); two-tailed p < 0.05 was considered statistically significant. The magnitude of between-group differences was assessed using Cohen’s d, implemented via the R effectsize package. Categorical data were processed in SPSS 20.0, presented as frequencies (percentages), and compared across groups using the chi-square test. The latent class growth model statistical analyses were performed using Mplus 8.0.

#### 2.5.2 Lipid data preprocessing and quality control

Among the 393 lipid metabolites detected (MRM parameters listed in Supporting document), we excluded all those with relative standard deviation (RSD) greater than 30% between QC samples or with a missing rate exceeding 30% across all samples. After screening, a total of 376 lipid metabolites meeting the quality requirements were retained for subsequent analysis. The lipidomics raw data were preprocessed using the Wukong platform, an R-based tool for omics data analysis (44). Lipid values were median-normalized, log2-transformed and scaled to 0 with SD 1. Missing values were imputed using random forest after median-normalization. Two lipid ratios were calculated: PE/diacyl-PC and ether-PC/diacyl-PC. The PE/diacyl-PC ratio was defined as the sum of PE species divided by sum of diacyl-PC among the six candidate lipids. The ether-PC/diacyl-PC ratio was calculated as the sum of ether-linked PC species (PC-O + PC-P) divided by the same sum of diacyl-PC. Both ratios were selected based on prior biological evidence: the PE/diacyl-PC ratio has been associated with insulin sensitivity (27), and the ether-PC/diacyl-PC ratio has been linked to glycemic dysregulation (45). To mitigate the impact of outliers, all ratios were log2-tansformed and scaled prior to statistical analysis. The PE/diacyl-PC ratio was primarily selected for statistical reasons: the conventional diacyl-PC/PE ratio exhibited extreme outliers (> 290 in controls, > 600 in hyperglycemia), and using its reciprocal (PE/PC) with log2-tranformation substantially compressed the distribution and reduced sensitivity to outliers.

#### 2.5.3 Lipid discovery based on FPG and covariate adjustment

To identify potential lipid metabolites associated with FPG from 376 candidates, we first applied simple linear regression for individual comparisons. P-values were adjusted using the Benjamini-Hochberg procedure for false discovery rate (BH) correction. Given the limited sample size, an BH-corrected p < 0.2 was used as the screening threshold to balance statistical power and error control, yielding a list of candidate lipid with suggestive significance.

To assess whether candidate lipids–FPG associations were independent of key confounders, each candidate lipids was entered into a separate multiple linear regression model. To guide the covariate selection strategy and visualize the hypothesized relationships among all study variables, we constructed a directed acyclic graph (DAG, **Supplementary Figure 1**) based on prior biological knowledge. In the DAG, solid arrows represent assumed causal directions, and the dashed line between lipidomics and FPG represents an associational link under investigation without a prespecified direction. All variables depicted in the DAG, including age, sex, BMI, early-life variables (income, maternal age, maternal pre-pregnancy BMI, gestational weight gain, birth ponderal index, gestational age, breastfeeding within 6 months, and growth trajectory at 0–2 years), conventional lipid parameters (TC, TG, HDL-C, LDL-C), and uric acid, were considered as covariates in the subsequent regression models.

Given the limited complete-case sample (n = 105), principal component analysis (PCA) was performed to reduce high-dimensional covariates. The first principle component (PC1)derived from early-life variables (including family income, AgeMum, mBMI, GWG, PI, gestational age, breastfeeding, and growth trajectory from 0 to 2 years) and from metabolic disorder variables (including HDL-C, LDL-C, TC, TG, and uric acid) were extracted as an ELR score and MD score, respectively. Theses scores, along with age, sex, and BMI, were included as covariates. The variance inflation factor (VIF) was then calculated for all variables in the final regression models to assess multicollinearity, and VIF values were < 5, indicating no substantial collinearity among the predictors. A Bonferroni-corrected *p* < 0.05 was considered statistically significant, identifying core lipids independently associated with FPG. The interaction between sex and core lipids (lipid × sex) was then examined in models adjusted for age, BMI, ELR score and MD score.

#### 2.5.4 Diagnostic potential evaluation based on categorical FPG

To develop a lipid-based diagnostic model for pediatric hyperglycemia, participants were classified into a normoglycemic control group (Ctr, FPG < 5.6 mmol/L) and a hyperglycemic group (HG, FPG ≥ 5.6 mmol/L). Using the full sample (n = 119), cross-validated LASSO regression was used for sparse feature selection, retaining lipids with non-zero coefficients. The top three lipids ranked with absolute coefficient magnitude were selected as high-confidence targets. The diagnostic performance of individual lipids and the three-lipid panel were evaluated using ROC curves with AUC and 95% confidence interval (95% CI) based on the same full sample (n = 119). Calibration of the three-lipid panel was assessed using the Hosmer-Lemeshow goodness-of-fit test, with p > 0.05 indicating adequate calibration.

To assess the incremental diagnostic value of the selected lipid panel beyond conventional clinical indicators, three nested logistic regression models were constructed using the subset with complete covariate data (n=105; hyperglycemia: n = 51): Model 1 included age z-score, sex; Model 2 added BMI z-score and ELI score; Model 3 further incorporated the three lipid species. The most complex model (Model 3) contained 7 predictors, yielding an events-per-variable (EPV) ratio of approximaterly 7.3 (51/7). To address potential overfitting given the moderate EPV, we employed a multi-pronged strategy: (1) LASSO regularization for variable selection; (2) bootstrap-based optimism correction (R = 2000) for internal validation; and (3) sensitivity analysis using Firth’s penalized logistic regression to reduce bias in small samples (46, 47). AUC differences between models were compared using DeLong’s test. Model calibrarion was assessed using the Hosmer-Lemeshow goodness-of-fit test for the logistic regression model containing the selected lipid species.

To test robustness of the lipid-based model, we performed a sensitivity analysis by adding the MD score to Model 3 (Model 3s), while retaining all other covariates. Additionally, to evaluate robustness against alternative case definitions, hyperglycemia was redefined using the 75th percentile of FPG (FPG ≥ 5.71 mmol/L, n = 119) distribution in this cohort as the new cutoff. The established diagnostic model was then reassessed for its diagnostic performance using this alternative case definition with LASSO regression (n = 105).

#### 2.5.5 Software

Analyses were performed using the following tools: PCA via Wukong platform (44), pathway analysis via MetaboAnalyst (www.metaboanlyst.ca), regression and receiver operating characteristic curves (ROC) analysis in R v4.3.1, and visualization in GraphPad 9.

### 2.6 Ethical approval

Written informed consent was obtained from all guardians prior to study procedures. The study protocol was approved by the Institutional Review Board (IRB) of Tianjin Women’s and Children’s Health Center (BGI-IRB 17116-201711).

## 3 Results

### 3.1 Physical and metabolic characteristics

**Table 1** presents the physical and metabolic characteristics of the participants. Compared with the Ctr group, the HG group showed significantly higher FPG and TG levels, as well as a higher BMI. Due to insufficient BMI records for three children (2 in Ctr, 1 in HG) under the age of 2 years, growth trajectories could not be calculated for these individuals. Thus, growth trajectories are only reported for 116 children (58 Ctr, 58 HG).

**Table 1:** Study population physical and metabolic characteristics*.

Variables	All [mean ± SD (n)]	HG [mean ± SD (n)]	Ctr [mean ± SD (n)]	Effect size† (95% confidence interval)	p
Age	3.83 ± 0.29 (119)	3.86 ± 0.32 (59)	3.81 ± 0.27 (60)	0.18 (-0.18, 0.54)	0.327
Sex:				0.008	0.926
female	60 (50.42%)	30 (50.85%)	30 (50.00%)		
male	59 (49.58%)	29 (49.15%)	30 (50.00%)		
BMI (kg/m^2^)	15.55 ± 1.31 (119)	15.85 ± 1.56 (59)	15.26 ± 0.95 (60)	0.46 (0.10, 0.83)	0.014
zBMI		0.23 ± 1.18 (59)	-0.22 ± 0.72 (60)		0.014
BF%	17.45 ± 5.02 (119)	18.00 ± 6.03 (59)	16.92 ± 3.75 (60)	0.22 (-0.15, 0.58)	0.244
Early-life risk variables					
Gestational age (weeks)	39.09 ± 1.28 (59)	38.98 ± 1.32 (58)	39.19 ± 1.24	-0.16 (-0.52, 0.20)	0.391
AgeMum(years)	28.86 ± 2.64 (57)	28.87 ± 2.37 (55)	28.85 ± 2.90	6.30e-03 (-0.36, 0.38)	0.974
ponderal index (g/cm^3^)	26.83 ± 2.66 (59)	26.72 ± 2.93 (58)	26.94 ± 2.39	-0.08 (-0.44, 0.28)	0.662
mBMI (kg/m^2^)	22.63 ± 3.53 (57)	23.29 ± 3.62 (55)	21.99 ± 3.34	0.37 (0.00, 0.74)	0.052
GWG (kg)	11.67 ± 3.74 (56)	12.33 ± 3.80 (54)	11.03 ± 3.60	0.35 (-0.03, 0.73)	0.068
Family income‡:				0.062	0.801
1	29 (24.79%)	15 (25.86%)	14 (23.73%)		
2	52 (44.44%)	24 (41.40%)	28 (47.46%)		
3	36 (30.77%)	19 (32.76%)	17 (28.81%)		
Breastfeeding:				0.113	0.224
1	44 (37.61%)	25 (43.10%)	19 (32.20%)		
2	73 (62.39%)	33 (56.90%)	40 (67.80%)		
0–2 year Growth tragectory§				0.117	0.453
Low	30 (25.86%)	14 (24.14%)	16 (27.59%)		
Middle	67 (57.76%)	32 (55.17%)	35 (60.34%)		
High	19 (16.38%)	12 (20.69%)	7 (12.07%)		
Metabolic disorder variables					
TC (mmol/L)	4.29 ± 0.48 (119)	4.37 ± 0.45 (59)	4.22 ± 0.50 (60)	0.31 (-0.05, 0.67)	0.090
HDL-C (mmol/L)	1.39 ± 0.22 (119)	1.38 ± 0.19 (59)	1.40 ± 0.26 (60)	-0.09 (-0.45, 0.27)	0.619
LDL-C (mmol/L)	2.34 ± 0.47 (119)	2.41 ± 0.47 (59)	2.28 ± 0.46 (60)	0.27 (-0.10, 0.63)	0.149
TG (mmol/L)	0.82 ± 0.25 (119)	0.91 ± 0.24 (59)	0.74 ± 0.23 (60)	0.73 (0.36, 1.10)	<0.001
FPG (mmol/L)	5.37 ± 0.68 (119)	5.92 ± 0.50 (59)	4.83 ± 0.30 (60)	2.66 (2.16, 3.15)	<0.001
UA (mmol/L)	217.82 ± 43.33 (119)	222.63 ± 42.74 (59)	213.10 ± 43.75 (60)	0.22 (-0.14, 0.58)	0.232

*Values are mean ± SD for continuous data and numbers (percentages) for categorical data. *P*-value was based on the independent samples t-test for continuous data and Chi-Square tests for categorical data.

HG, Hyperglycemia group; NC, normoglycemia control group; BF%, Body fat percentage;BMI, body mass index; zBMI, BMI z-scores; FPG, fasting plasma glucose; TG, triglyceride; TC, total cholesterol; LDL-C, low-density lipoprotein–cholesterol; HDL-C, high-density lipoprotein–cholesterol; UA, uric acid; AgeMum, mother’s age when gave birth to baby; GWG, Gestational Weight Gain; mBMI, Pre-pregnancy BMI;

† Effect size: For continuous variables, effect sizes and their confidence intervals were calculated using Cohen’s d. For categorical variables, effect sizes were derived from the φ (phi) coefficient of the chi-square test.

‡ family income:: 1: income < ¥100,000; 2: income between ¥100,000 and ¥200,000; 3: income >= ¥200,000.

Breastfeeding: 1: Non-breastfed for the first 6 months; 2: Breastfed for the first 6 months;

§ Growth trajectory:.

Low, Low-trajectory group; BMI z values ranging from -0.5 to 0;

Middle, Middle-trajectory group; BMI z values ranging from 0 to 0.5;

High, High-trajectory group; BMI z values ranging above 0.5.

### 3.2 Dimensionality reduction and construction of composite scores

To ensure analytical quality, samples with missing data on key baseline variables have been excluded. The PC1 of early-life variables accounted for 23.8% of the total variance (**Supplementary Figure 2A**). Although modest, PC1 captured the most significant shared pattern (**Supplementary Table 2**), with loadings defined a biologically plausible “overall adverse early-life environment” profile characterized by positive associations with PI, gestational age, and breastfeeding, and a negative association with maternal age (**Supplementary Table 3**). The PC1 scores were thus used as a composite “Early-Life Risk Score” in all subsequent regression analyses to adjust for early-life confounding.

Similarly, PC1 from metabolic variables explained 38.3% of variance (**Supplementary Figure 2B**, **Supplementary Table 2**) and was characterized by positive loadings for LDL-C and TC (**Supplementary Table 4**). This component was retained as a Metabolic Disorder Score and included as a covariate in all subsequent regression models.

### 3.3 Screening and characteristic analysis of lipid metabolites significantly associated with FPG

#### 3.3.1 Screening of lipid metabolites significantly correlated with FPG in global lipid profiling

**Figure 1:**
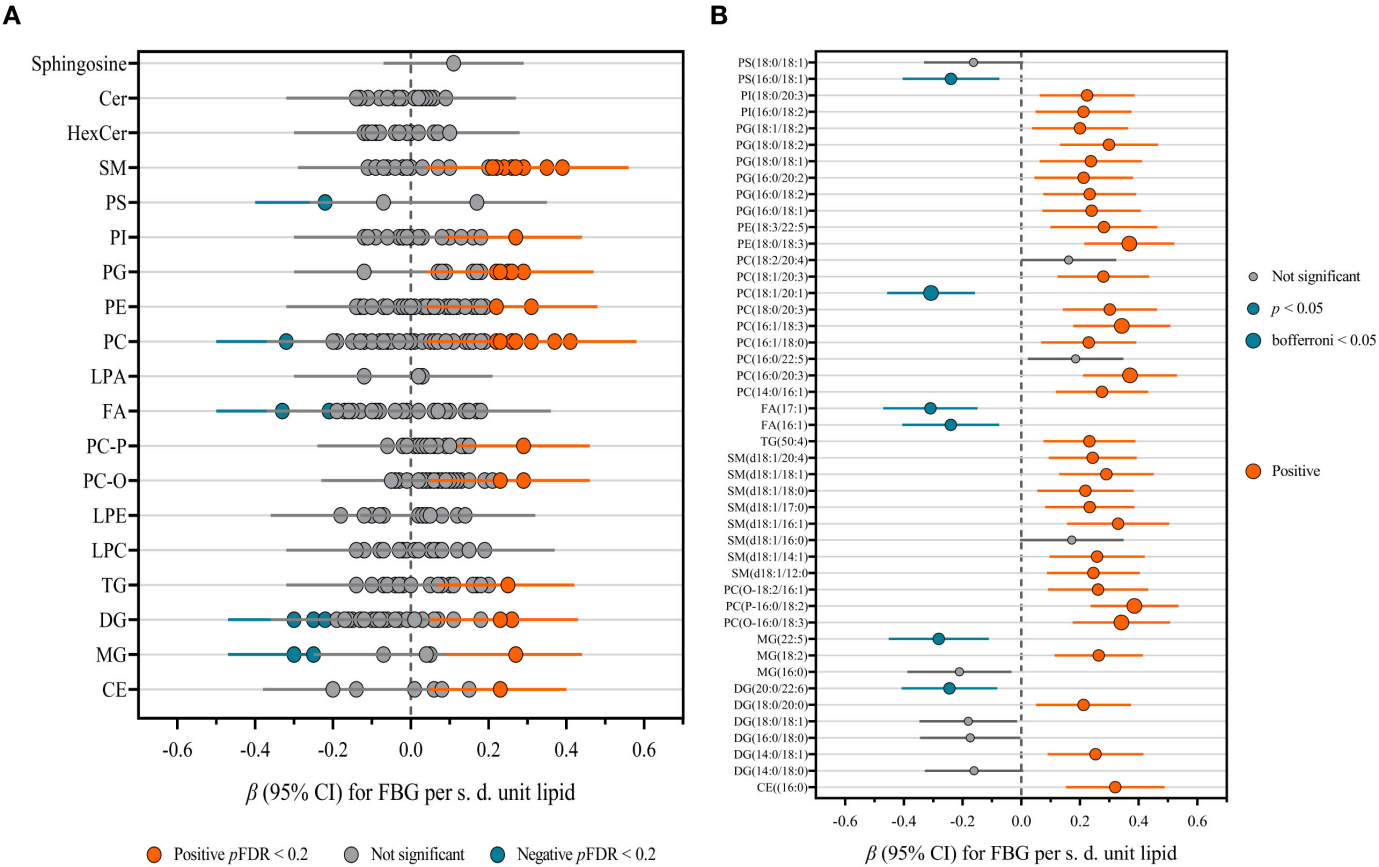
Overview of the plasma lipidome associated with FPG. **(A)** Associations between 376 lipids and FPG in all preschool-aged children using a simple linear regression. **(B)** Associations between 45 lipids and FPG with multiple linear regression.

To identify key lipids associated with FPG in children, we first conducted a systematic analysis of the 376 lipids included in the study. Preliminary univariate linear regression analysis revealed that 67 lipids were nominally associated with continuous FPG levels (p < 0.05, **Supplementary Table 5**; **Figure 1A**). After BH_FDR correction, 45 lipids showed suggestive significant associations at an BH_FDR threshold of less than 0.2 (**Supplementary Table 5**, **Supplementary Figure 2A**).

To examine whether these associations were independent of other influencing factors, we incorporated these lipids into multiple linear regression models adjusted for age, sex, BMI, ELR score and MD score. To maintain analytical rigor, all samples with incomplete baseline data were excluded due to missing values in key variables. As a result, subsequent multivariate analyses were conducted on a final cohort of 105 samples with complete baseline data. The results indicated that 37 lipids remained significantly associated with FPG levels at p < 0.05. Among these, 6 lipids showed significant negative correlations with FPG, while 31 exhibited significant positive correlations (**Figure 1B**). Lipids that met the Bonferroni-corrected threshold of p < 0.05 were defined as core lipids for subsequent analyses. Of the 6 core lipids, three belonged to phosphatidylcholines (PCs), two were alkyl-PCs, and one was a phophatidylethanolamine (PE). Pathway analysis revealed that all six lipids belong to the glycerophospholipid metabolism pathway (**Supplementary Figure 3**).

#### 3.3.2 Association of core glycerophospholipid subtypes and metabolic ratios with FPG

To characterize the disturbance of glycerophospholipids from the perspective of overall metabolic homeostasis, four biologically meaningful glycerophospholipid metabolic ratios were constructed based on six core lipid species, including three diacyl-PC, two ether-linked PCs (one PC(O), and one PC(P)), and one PE. The derived ratios included PE/PC (diacyl-PC), ether-linked PC/PC (diacyl-PC).

**Figure 2:**
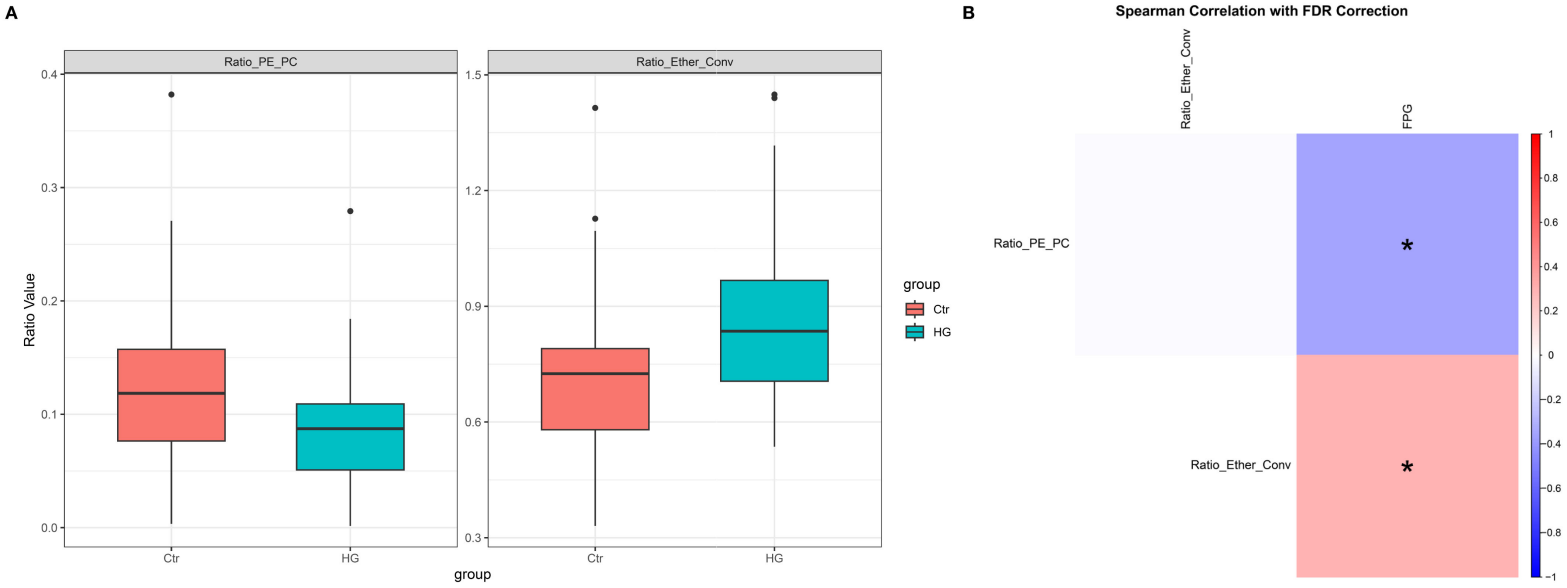
Comparison of four glycerophospholipid metabolic ratios and their correlation with FPG. **(A)** Box plots of the two glycerophospholipid ratios between the control (Ctr) and hyperglycemia (HG) groups. **(B)** Correlation heatmap showing the associations between the two glycerophospholipid ratios and FPG.

Group distribution and difference analyses showed that PE/PC, ether-linked PC/PC differed significantly between the Ctr and HG groups (P < 0.05) (**Figure 2A**). Further Spearman correlation analysis and multivariate linear regression adjusted for age z-score, sex, and BMI z-score demonstrated that PE/total PC (β = -0.29, bonferroni-p < 0.05) were independently and negatively associated with standardized FPG (**Figure 2B**, **Table 2**). In contrast, the ether-linked PC/PC ratio(β = 0.37, bonferroni-p < 0.05), which presented independent and positive correlation with FPG (**Figure 2B**, **Table 2**).

**Table 2:** Multivariate linear regression analysis of two glycerophospholipid metabolic ratios in relation to FPG*.

Ratio	Beta	P_value
Ratio_PE_PC	**4.9965**	**0**
Ratio_Ether_Conv	-0.0034	0.1342

*Models were adjusted for age, sex, Z-standardized BMI, Early-Life Risk Score, and Metabolic Disorder Score. Bold values indicate *P* < 0.05.

These findings indicated that both the overall PE/PC balance and the relative abundance of ether-linked PC over conventional diacyl PC were independently associated with FPG levels in this pediatric cohort, albeit in opposite directions.

### 3.4 Sex differences in lipid-FPG associations

To investigate whether the associations between core lipids and FPG were sex-specific, we introduced a “lipid × sex” interaction term into models adjusted for age, BMI, ELR score, and MD score. Among the six core lipids tested, only one lipid (PC(16:1/18:3)) showed a nominally significant interaction with sex (p = 0.0276, **Supplementary Table 6**). Stratified analysis revealed a strong positive association between PC(16:1/18:3) and FPG in boys (*β* = 0.501, p < 0.001), whereas no significant association was observed in girls (*β* = 0.09, p= 0.5709).

For the remaining five lipids, no significant sex differences in their associations with FPG were detected (all interaction p > 0.05, **Supplementary Table 6**). As only one isolated significant signal was observed, which did not survive multiple comparison correction (BH_FDR>0.05, **Supplementary Table 6**), our study did not find sufficient evidence to support widespread sex differences in lipid-FPG associations. Therefore, the primary conclusions are based on analyses and results derived from the full sample.

### 3.5 Diagnostic value of the lipid panel for pediatric hyperglycemia

As shown in the LASSO coefficient path plot (**Supplementary Figure 4A**), variable coefficients gradually converged to zero with increasing regylarization parameter λ. The optimal λ (λ.1se) was selected based on cross-validation error (**Supplementary Figure 4B**). Under this selected model, five lipids retained non-zero coefficients (**Supplementary Figure 4C**). The top three lipid species were selected according to the absolute magnide of their regression coefficients, PC(P-16:0/18:2), PC(16:0/20:3), and PC(16:1/18:3) were identified as the most influential lipids and were carried forward for further analysis. The discriminative performance of this three lipid panel for pediatric hyperglycemia (FPG ≥ 5.6 mmol/L) was subsequently evaluated.

To evaluate the independent associations of lipid metabolites with disease risk, we constructed stepwise-adjusted Logistic regression models (**Table 3**). PC(P-16:0/18:2), PC(16:0/20:3), and PC(16:1/18:3) were significantly associated with disease risk across all models (FDR-adjusted q < 0.05). In the primary Model 3 (adjusted for age z-score, sex, and BMI z-score, n = 105), the ORs were 2.34 (95% CI: 1.39-4.32), 3.58 (1.96-7.37), and 3.07 (1.74-5.98), respectively (all q<=0.05). Male sex, with male sex showeing a protective effect (OR = 0.06, p = 0.002) and BMI z-score being marginally significant (OR = 2.06, p =0.053). In sensitivity analysis, further adjustment for MD score increased the ORs for all three lipids (PC(16:1/18:3) showed a 2.5-fold increase), with MD score itself independently associated with disease risk (OR = 3.17, p < 0.001). The enhanced lipid effects after MD score adjustment argue against confouding and suggest a potential additive effect, further confirming the robustness of the observed associations.

**Table 3:** Odds ratios (Ors) and significance of three PC metabolites and the covariables in multivariable logistic regression models.

Variable	Model 1*		Model 1b*		Model 2*		Model 3*	
	OR (95% CI) †	P_fdr‡	OR (95% CI) †	P_fdr‡	OR(95% CI) †	P_fdr‡	OR(95% CI) †	P_fdr‡
PC(P-16:0/18:2)	1.96(1.24-3.32)	0.0408			2.25(1.35-4.13)	0.04	2.34(1.39-4.32)	0.0348
PC(16:0/20:3)	2.47 (1.53-4.34)	0.0036			3.49(1.961-6.95)	0.001	3.58(1.96-7.37)	0.0012
PC(16:1/18:3)	2.20(1.36-3.80)	0.0144			3.03(1.76-5.67)	0.002	3.07(1.74-5.98)	0.0036
z-age§			1.22(0.84-1.78)	1	0.96(0.58-1.571	1	0.96(0.57-1.58)	1
sexmale			0.89(0.42-1.86)	1	0.19(0.06-0.55)	0.038	0.17(0.05-0.52)	0.042
zBMI§§							1.82(1.10-3.30)	0.3804

*Model 1: multivariable logistic regression models including three PC lipids; Model 1b: multivariable logistic regression models including age z-score, sex; Model 2: multivariable logistic regression models including age z-score, sex, and the three PC lipids; Model 3: multivariable logistic regression models including z-age, sex, zBMI, and the three PC lipids.

† OR, odds ratio; CI, confidence interval. ORs were derived from univariate logistic regression models.

‡ P_fdr: P values were adjusted for multiple comparisons using the Benjamini-Hochberg false discovery rate (FDR) method to control the expected proportion of false positives among the three PC species tested.

§z_age, age z-score.

§§zBMI, BMI z-score.

We next evaluated the individual discriminative performance of each lipid using ROC curve analysis. The AUC values for PC(P-16:0/18:2), PC(16:0/20:3), and PC(16:1/18:3) were 0.653 (95% CI: 0.555-0.749), 0.721 (95% CI: 0.626-0.810) and 0.691 (95% CI: 0.594-0.777, **Supplementary Figures 5A–C**). We further visualized the between-group distribution of the three PC species in hyperglycemic versus control children (**Supplementary Figures 5D–F**), which showed consistently higher levels in the hyperglycemic group across all three PC species (all p < 0.05, Wilcoxon rank-sum test). These findings suggest that each of the three lipids individually exhibits moderate discriminative ability for pediatric hyperglycemia.

**Figure 3:**
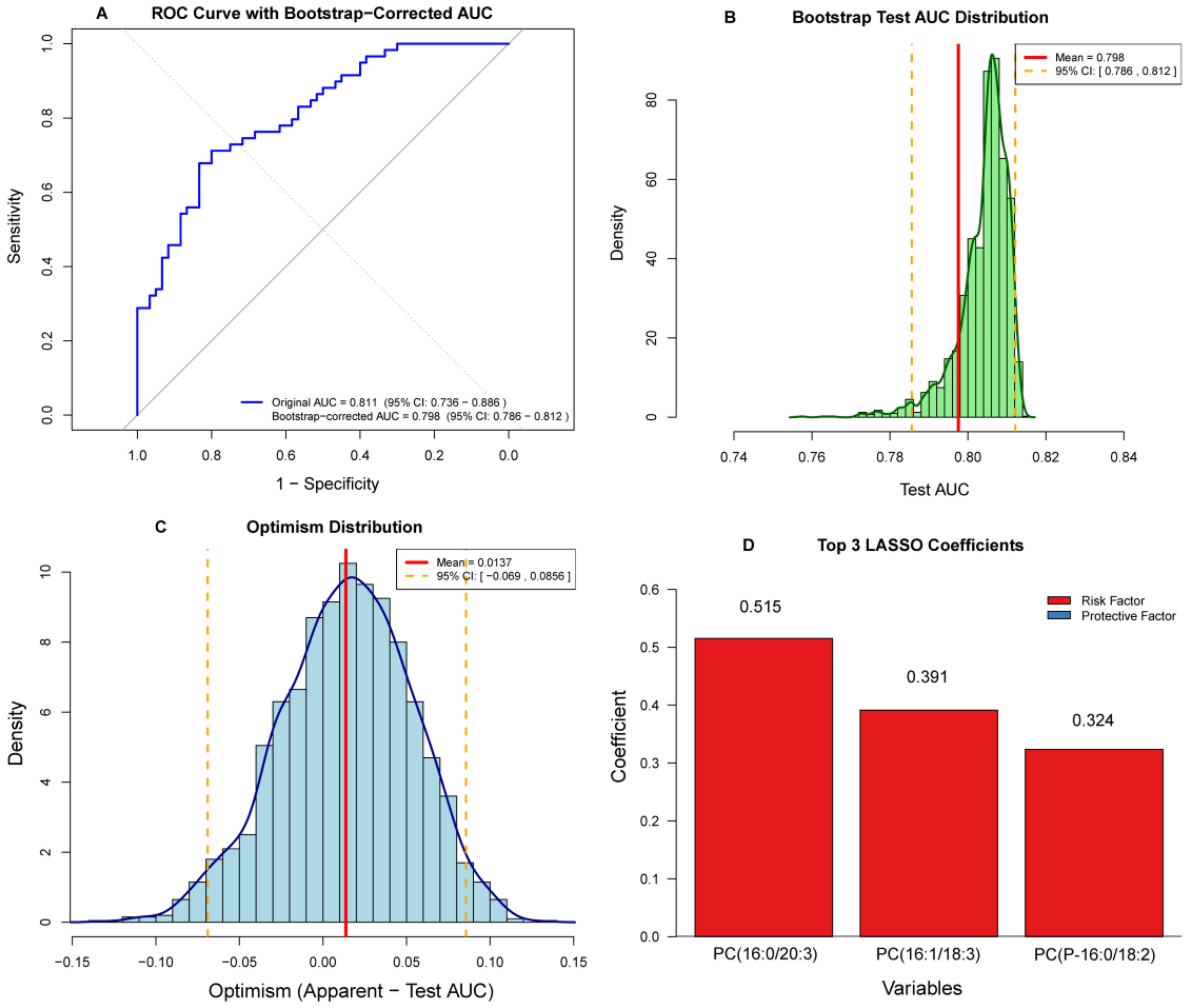
Relative contribution of three lipid species to the diagnosis of pediatric hyperglycemia. **(A)** Receiver operating characteristic (ROC) curve of the diagnostic model with three LASSO-selected lipids, namely, PC(P-16:0/18:2), PC(16:0/20:3), and PC(16:1/18:3). **(B)** Bootstrap test AUC distribution of 2000 replicates. **(C)** Optimism distribution of bootstrap test. **(D)** The bar plot of three PC species' coefficients selected by LASSO.

To assess the diagnostic performance of this lipid panel, we constructed a multivariate logistic regression model incorporating only these three lipids. The original logistic regression model yielded an AUC of 0.811 (95% CI: 0.733–0.881). Bootstrap analysis with 2000 resamples yielded an average optimism of 0.0136 (**Figure 3C**). After bias correction, the model achieved an AUC of 0.798 (Bootstrap 95% CI: 0.786–0.812) (**Figures 3A, B**), suggesting that this lipid panel demonstrates good discriminative ability for hyperglycemia.

The calibration curve showed good agreement between predicted probabilities and observed outcomes, supported by a non-significant Hosmer-Lemeshow test (p > 0.05), indicating satisfactory model calibration. In addition, the negligible optimism (0.0136) from 2000 bootstrap resamples suggested that the model was not substantially overfitted.

Collectively, these results demonstrated that a three-lipid panel comprising PC(P-16:0/18:2), PC(16:0/20:3), and PC(16:1/18:3) is independently associated with pediatric hyperglycemia and exhibits robust discriminative performance, supporting its potential as a candidate lipid panel.

### 3.6 Incremental diagnostic value of the lipid panel beyond conventional clinical parameters

To further evaluate the incremental diagnostic value of the lipid panel beyond established risk factors, this study systematically compared the diagnostic performance of three nested models.

**Figure 4:**
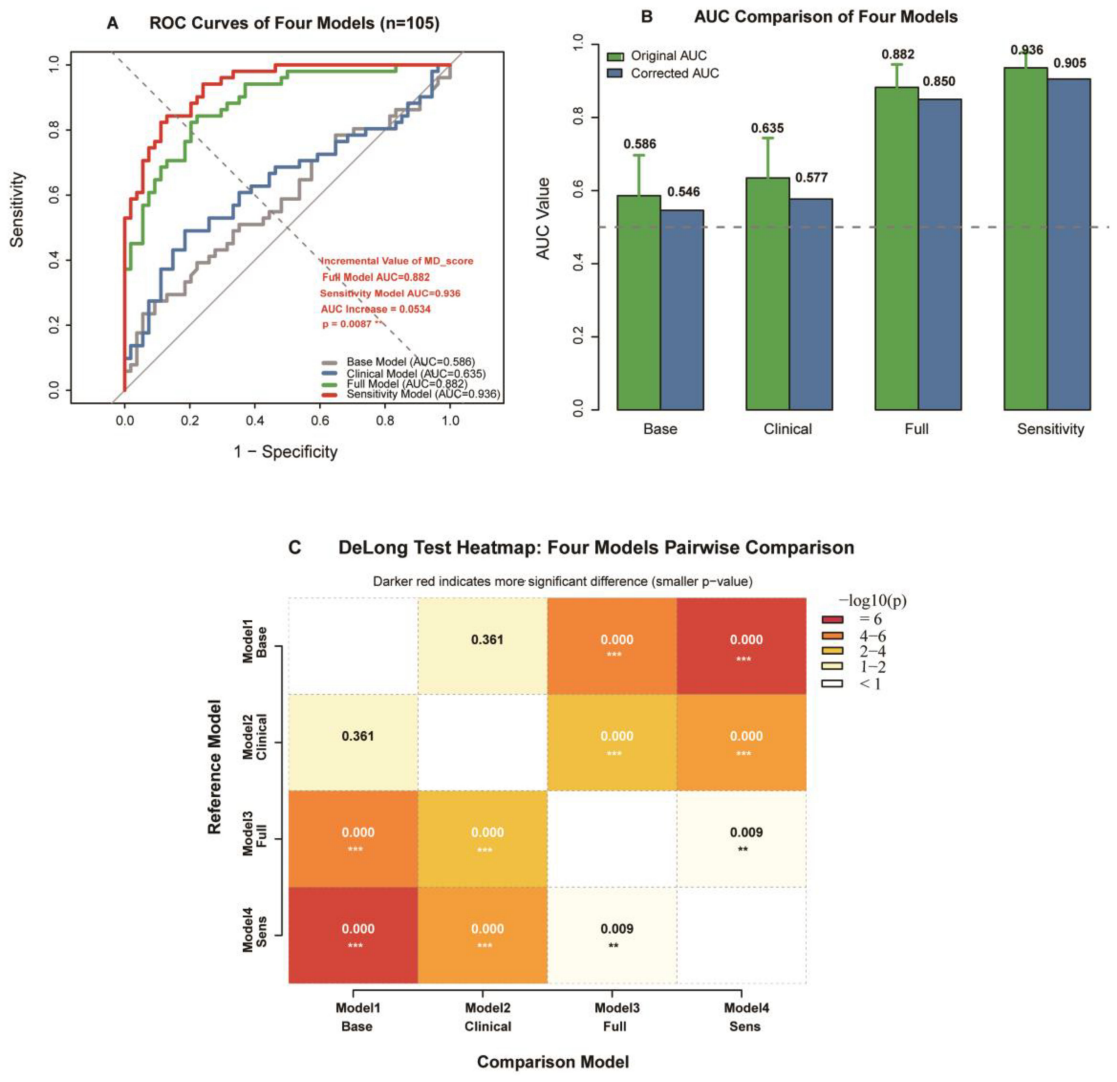
Comprehensive performance of three models. **(A)** The ROC curve when fitted values under the three models. **(B)** The original and bootstrap corrected AUCs of the three models. **(C)** Comparison of AUC under the three models with DeLong’s test.

First, we constrcted a baseline model (Model 1) that included only age, sex. After internal validation via bootstrapping, Model 1 achieved an AUC of 0.586 (corrected AUC = 0.546, 95% CI: 475-0.697, **Figure 3A**). Next, by incorporating BMI z-score and early-life risk score into Model 1, we developed an extended clinical model (Model 2), which yielded an AUC of 0.635 (corrected AUC = 0.577, 95% CI: 0.525–0.744, **Figure 4A**). Compared with Model 1, Model 2 showed an AUC increase of 0.049. However, this improvement was not statistically significant (DeLong’s *p* = 0.361, **Figure 4B**). Finally, building upon Model 2, we further integrated the lipid panel (PC(P-16:0/18:2), PC(16:0/20:3), PC(16:1/18:3)) to established a comprehensive model (Model 3). This model demonstrated a marked enhancement in discriminative performance, with a corrected AUC of 0.882 (corrected AUC = 0.850, 95% CI: 0.820-0.945) (**Figure 4A**). Furthermore, the sensitive model by intergrating MD score to Model 3 (Model 3s) to assess the effect of MD score. The results showed the AUC was 0.936 (corrected AUC = 0.905, 95% CI: 0.894-0.978, **Figure 4A**).

Pairwise model comparisons using DeLong’s test revealed that Model 3 demonstrated a significant improvement in diagnostic performance compared with Model 1, with a ΔAUC of 0.296 (DeLong’s *p* < 0.001, **Figure 4B**). Similarly, Model 3 also showed a statistically significant enhancement relative to Model 2, with a ΔAUC of 0.248 (DeLong’s *p* < 0.001, **Figure 4B**). To assess the robustness of our findings given the moderate events per variable (EPV = 6.4), we conducted a sensitivity analysis using Firth’s penalized logistic regression. The Firth-penalized model produced AUCs that were virtually nearly identical to the primary models across Model 3 (AUC: 0.882 vs. 0.883) and Model 3s (AUC: 0.936 vs. 0.935) with all differences below 0.002. This near-perfect concordance confirms that our results are robust and not materially influenced by the moderate EPV or potential separation issues.

Sensitivity analysis.

Beyond the estimation method, we also assessed the generalizability of our findings to alternative diagnostic criteria for hyperglycemia. To assess the robustness of our findings to the choice of FPG cutoff, we re-run the analysis using the 75th percentile of the study population’s FPG distribution (P75 = 5.71 mmol/L) as an alternative definition of hyperglycemia. To avoid overfitting given the limited number of cases (n = 27 at the P75 cutoff), LASSO regression with 10-fold cross-validation was employed for variable selection (lambda.1se), which identified three variables (BMI z-score, PC(16:0/20:3), PC(16:1/18:3)). In this sensitivity analysis, the LASSO-selected model demonstrated an AUC of 0.810 (95% CI: 0.722-0.898), significantly higher than that the clinical model (AUC: 0.834 vs 0.670), with ΔAUC of 0.165 (DeLong’s test, p = 0.0119) (**Supplementary Figure 6**). This confirms the incremental diagnostic value of lipid panel.

These findings are consistent with the primary analysis using the clinical cutoff (ΔAUC = 0.247, p < 0.001), indicating that the incremental value of the three lipids is robust and not dependent on the specific definition of hyperglycemia.

## 4 Discussion

This cross-sectional lipidomics study identified three key PC species, PC(P-16:0/18:2), PC(16:0/20:3), PC(16:1/18:3), as independent markers associated with subclinical hyperglycemia (FPG ≥ 5.6 mmol/L) in preschool-aged children. The three-PC lipid panel demonstrated robust discriminative capacity (AUC = 0.882, bias-corrected AUC = 0.850, 95% CI: 0. 820-0.945), and significantly outperformed models based on conventional clinical indicators alone (ΔAUC = 0.247, p < 0.001). This performance remained stable in sensitivity analyses using Firth penalized regression (AUC = 0.883) and an alternative FPG cutoff (75th percentile, AUC = 0.834), supporting the robustness of our findings.

Our findings advance the field in three specific ways. First, this study focuses specifically on preschool-aged children, an age group that has largely overlooked in prior metabolic lipidomic research. Second, by focusing on normal-weight children with subclinical hyperglycemia rather than overt disease, we identified early metabolic signatures associated with glycemic elevation before clinical diagnosis, which may inform early detection strategies. Third, the combined PC panel provides a practical, multi-lipid composite score with potential utility as a screening indicator for metabolic risk in children.

### 4.1 Diagnostic performance and biological relevance of biomarkers

Current clinical markers, such as FPG, lack specificity and sensitivity for early diabetes detection (48). In this context, increased levels of PC(P-16:0/18:2), PC(16:0/20:3), and PC(16:1/18:3) were found to be significantly and positively associated with FPG, consistent with previous evidence in adults (49) and children aged 7–17 years old (20). In a prospective, nested case-control study within the EPIC-Potsdam cohort, Anna Floegel et al. (49) enrolled 27,548 adults and employed tandem mass spectrometry to analyze 800 incident T2D cases and a random subcohort of 2,282 participants. Their findings revealed that each 1-SD increase in baseline diacyl-PCs (C32:1, C36:1, C38:3, C40:5) was associated with increased risk of T2D (relative risk (RR) range: 1.29-1.48), while the acyl-alkyl-PCs (C34:3, C40:6, C42:5, C44:4, C44:5) were inversely associated with T2D risk (RR range: 0.72-0.78), highlighting the distinct roles of PC subclasses in glucose metbaolism. Beyond adult populations, similar lipidomic signature have also been observed in pediatric cohort. In a cross-sectional study utilizing non-targeted lipidomics, plasma lipid profiles were compared among children with type 2 diabetes (aged 11–16 years), metabolic syndrome (MetS, aged 10–15 years), and healthy controls (aged 7–14 years) (20). The results demonstrated that elevated levels of PCs, SMs, and TAGs were positively correlated with multiple metabolic risk indicators, including BMI, waist circumference, blood glucose, insulin, and HOMA-IR (p < 0.05). However, the age ranges varied considerably across the three subgroups, with healthy controls being notably younger than the clinical groups, a factor that may independently influence lipid metabolism and confound between-group comparisons. Despite this limitation, the overall directional consistency across adult and pediatric studies supports the potential role of these PC species as early metabolic markers, corroborating our finding that specific PCs are positively correlated with FPG.

The fact that two of the three identified PC species contain palmitic acid (C16:0) aligns with prospective evidence from the EPIC study, where elevated saturated fatty acids (C16:0 and C18:0) in plasma phospholipids were associated with the increased T2D incidence in adults (hazard ratio per SD: 1.26 [1.15–1.37] for C16:0) (6). Notably, elevated PC(16:1/18:3) (PC 34:4) levels showed the strongest association with childhood hyperglycemia (defined as FPG≥5.6mmol/L), with each 1-unit increase in log-transformed PC(16:1/18:3) corresponding to an odds ratio of 3.49 (95% CI: 1.96-6.95). Previous adult studies showed PC(34:4) levels were associated with insulin resistance (50). Lehmann et al. (50) employed targeted metaboliomics to demonstrat that decreased concentrations of PC(aa 34:4) were associated with insulin resistance in patients with gestational diabetes mellitus (GDM). In a cohort of 472 community-dwelling older adults (mean age 70.7 years) from the Baltimore Longitudinal Study of Aging, Semba et al. (22) employed targeted metabolomics and found that plasma levels of PC(aa C34:4) were significantly associated with lower odds of IFG and insulin resistance (HOMA-IR). These results in adults were in contrast with our positive association in pediatric populations, indicating that their pathological significance may vary across life stages.

Two lipid ratios, PE/diacyl-PC and ether-PC/diacyl-PC, were independently associated with FPG in opposite directions (**Figure 2A**). The inverse association between PE/PC, i.e. lower PE relative to PC, correlating with higher glucose, is directionally consistent with previous evidence from skeletal muscle studies, where a higher PC/PE ratio (equivalent to lower PE/PC) was associated with diminished insulin sensitivity in adults (27). Jonscher et. al (51). reported that, in a mouse model of maternal Western-style diet, pyrroloquinoline quinine supplementation reduced the hepatic PC/PE ratio in offspring and concurrently improved their glucose tolerance. The above findings at the tissue level are biologically consistent with our observations in plasma, where a decreased PE/PC ratio (i.e., increased PC/PE) was associated with an elevated risk of hyperglycemia, suggesting that the regulatory role of PE/PC balance in glucose metabolism may be conserved across different tissues. Mechanistically, PC enrichment in membrane bilayers favors membrane fluidity (52), whereas PE enrichment decreases it, while membrane fluidity could affect the binding affinity of insulin to its receptor binding affinity, and downstream signal transduction efficiency (40, 53). Thus, our plasma-based observation of lower PE/PC in children with higher FPG suggests that PE depletion, rather than PE enrichment, may be linked to impaired glucose metabolism in pediatric population.

We initially examined the PC/PE ratio, which is more commonly reported in the literature. However, PC/PE ratio exhibited extremely high values in both groups (e. g. > 290 in the Ctr group and > 600 in the hyperglycemia group). Nevertheless, even with these extreme values, the ratio maintained a robust independent positive association after multivariate adjustment and sensitivity analysis (**Supplementary Figure 5**). To mitigate the influence of extreme values on regression estimates, we concurrently employed the PE/PC ratio (the reciprocal of PC/PE), which effectively compressed the data distribution range and enhanced model robustness. This comparison underscores the importance of intergrating distributional characteristics, outlier identification, and multivariate association analyses to distinguish robust biological signals from statistically driven artifacts.

Notably, the ether-PC/diacyl-PC ratio also differed significantly between groups (p = 0.0004), and showed a significant positive correlation with FPG, remaineding independently associated with FPG after covariate adjustment. This suggests that, in our pediatric cohort, a relative enrichment of ether-linked PC is associated with higher fasting. This finding contrasts with previous large-scale lipidomic studies in adults, which reported significant negative associations between ether-linked phospholipids (alkyl-PC and alkenyl-PC) and both prediabetes and type 2 diabetes (45). This discrepancy may reflect age-specific differences in lipid metabolism or pathophysiology, as our study population comprises preschool-aged children, whereas prior work has focused on adults. The positive association we observed could also suggest a developmental or compensatory response in early-life glycemic regulation that differs from the established paradigm in adults. Overall, these findings indicate that glycerophospholipid homeostasis may play a role in glycemic regulation in children, but the direction and mechanisms of these associations warrant further investigation, particularly given the contrasting evidence from adult populations.

### 4.2 Incremental value and clinical translation potential of lipid panel

The finding of this study regarding the incremental diagnostic value of specific phosphatidylcholine (PC) combinations aligns with recent advances in the pediatric lipidomics field and provides novel additions to the existing evidence. One study employing a Mendelian randomization design identified phosphatidylcholine ae C42:3 as one of the most promising biomarkers for young-onset type 2 diabetes and validated its association with childhood dysglycemia in a longitudinal cohort (54). Another study reported that a specific PC species (18:1e_16:0) achieved an AUC of 0.80 for diagnosing insulin resistance in children aged 7–may be conserved across different tissues 12 years, significantly outperforming traditional lipid parameters such as triglycerides, total cholesterol, HDL-cholesterol, and LDL-cholesterol (55).

Distinct from these studies that emphasized single PC species, our study found that individual PC species exhibited only modest diagnostic ability, whereas a combination of three specific PC species produced a marked synergistic enhancement. On top of traditional risk factors (age, sex, BMI, and early-life risk score), this three-lipid model increased the AUC by 0.247 (from 0.635 to 0.882, DeLong’s test p < 0.001), indicating its capacity to capture additional information on glycemic dysregulation. The robustness of our lipidomic model was further supported by two independent validation approaches: Firth penalized regression, which accounts for rate-event bias, confirmed consistently high diagnostic performance, and sensitivity analysis using the 75th-percentile FPG cutoff yielded similar robust AUC (> 0.80). To our knowledge, no previous pediatric lipidomics studies have quantified such incremental utility in preschool-aged children. In an adult-related study on combined lipid biomarkers for early detection of prediabetes and type 2 diabetes, an 8-lipid panel including LPC, PC, PE, two ceramide/SM ratios, and three TAGs achieved an AUC of 0.841 for prediabetes and 0.894 for type 2 diabetes, but this research only reported odds ratios in the validation cohort, yet the AUC of this panel was not reported (25). In pediatric research, a cord-blood study from Finland’s Type 1 Diabetes Prediction and Prevention (DIPP) cohort indicated that a signature comprising four TAGs and one cholesterol ester predicted the risk of progression to type 1 diabetes with an AUC of 0.83 (30). However,neither of these studies evaluated the incremental diagnostic value of lipid biomarkers beyond conventional risk factors. Moreover, adult-derived findings cannot be directly extrapolated to children, given the distinct developmental features of lipid metabolism during early life. Our study addresses these gaps by providing, to our knowledge, the first evidence that lipid panel offer significant incremental diagnostic value beyond conventional risk factors for identifying hyperglycemia in preschool-aged children.

### 4.3 Interpretive considerations regarding PC metabolism

The present lipidomics analysis is biologically anchored in the well-recognized crosstalk between FPG and lipid metabolism, wherein hyperglycemia drives lipid remodeling through pathways such as *de novo* lipigenesis, insulin resistanc-associated lipolysis, and glycation-related enzyme dysregulation (56–59).Within this framework, our finding that PC species exhibited a significant positive correlation with FPG and conferred incremental diagnostic value for hyperglycemia is mechanistically coherent. PC is the most abundant phospholipid in cellular membranes, and its metabolic homeostasis is tightly governed by the balance between PLA2-mediated hydrolysis and PEMT-mediated synthesis (44–48).Hyperglycemia-induced oxidative stress is known to activate PLA2, promoting the hydrolysis of PC into LPC and free fatty acid (44, 45). This process not only compromises membrane fluidity and integrity but also generates bioactive lipid intermediates that act as second messengers to activate pro-inflammatory cascades (e.g. NF-κB) (58, 60, 61), further exacerbating insulin resistance (60). Therefore, the elevated circulating PC levels observed in our hyperglycemic group may not simply reflect increased synthesis, but rather a compensatory accumulation resulting from disrupted hydrolytic flux under chronic hyperglycemic stress. This hypothesis is indirectly supported by existing evidence. A study published in 2001 demonstrated that high extracellular glucose concentrations (25 mM) directly increased the *de novo* synthesis of phosphatidylcholine in renal papillary cells of control rats. Moreover, in chronically diabetic rats, high glucose stimulation exerted an additive effect on top of the diabetes-induced elevation (62). Furthermore, PC serves as a critical precursor for cardiolipin synthesis in the inner mitochondrial membrane, where cardiolipin is essential for maintaining the structural integrity of the electron transport chain and mitochondrial bioenergetics. Disruption of PC homeostasis may therefore have downstream consequences for mitochondrial function, a pathway increasingly recognized in the pathophysiology of insulin resistance and hyperglycemia. A study using a type 1 diabetes animal model confirmed that short-term (4-week) hyperglycemia is sufficient to induce phospholipid remodeling in cardiac mitochondria, characterized by increased PC levels, accompanied by enhanced cardiolipin peroxidation and decreased oxidative phosphorylation (OXPHOS) function (63). The additional observation that the etherPC/diacylPC ratio was associated with hyperglycemia further supports the oxidative stress framework, as ether phospholipids are particularly susceptible to oxidative damage due to their labile ether bonds, and their relative abundance may serve as an indicator of redox imbalance in the setting of hyperglycemia.

### 4.4 Study strengths and limitations

Key strengths of this study include the targeted lipidomics approach with rigorous quality control, and multiple sensitivity analyses (Bootstrap validation, Firth penalized regression, alternative outcome definition), all of which strengthen the reliability of the findings. Nevertheless, several limitations should be noted. First, the cross-sectional design precludes causal inference. Although our lipid measurements were conducted prior to the diagnosis of hyperglycemia within this cross-sectional framework, reverse causation cannot be entirely ruled out, for instance, subtle glycemic excursions may have already influenced lipid metabolism before the single time-point assessment. Second, although internal validation was conducted, the sample size remains relatively limited, and external validation in larger independent prospective cohorts is warranted. Third, the incremental value of the lipid panel should be interpreted cautiously. The clinical model showed only moderate performance (AUC = 0.635) in this preschool-aged cohort, partly due to the limited variability of traditional indicators such as BMI at this age. Therefore, the ΔAUC of 0.247 upon addition of the lipid panel may be partially inflated and warrants external validation. Finnaly, residual confounding from unmeasured lifestyle factors, such as dietary pattern and physical activity, could not be fully eliminated despite adjustment for major available covariates.

## 5 Conclusion

In this cross-sectional targeted lipidomics study of preschool-aged children, three PC species, PC(P-16:0/18:2),PC(16:0/20:3), and PC(16:1/18:3), were independently associated with subclinical hyperglycemia (FPG ≥ 5.6 mmol/L). The lipid panel comprising these three PCs demonstrated significantly superior diagnostic value over conventional clinical indicators, with ΔAUC of 0.247 (p < 0.001) and a bias-corrected AUC of 0.850. Sensitivity analyses using Firth’s penalized logistic regression and alternative FPG cutoff (75th percentile, 5.71 mmol/L) confirmed the robustness of the panel’s diagnostic performance. In addition, two lipid ratios, PE/diacyl-PC and ether-PC/diacyl-PC, were also independently associated with FPG. These findings identify noval lipid panel associated with hyperglycemia in preschool-aged children, offering a potential diagnostic signature for current glycemic status, while also extending the investigation of early glycemic metabolic disturbances to the preschool-aged period, a critical window for metabolic programming that has been understudied. Future work should validate this lipid panel in independent cohorts and conduct longitudinal studies to clarify its prognostic utility for disease progression, thereby supporting the development of targeted prevention strategies in pediatric populations.

## Data Availability

The raw data supporting the conclusions of this article will be made available by the authors, without undue reservation.

## Glossary

FPG: fasting plasma glucose
GLPs: glycerophospholipids
GLs: glycerolipids
SLs: sphingolipids
SM: sphingomyelins
TG: triglyceride
HG: hyperglycemia group
Ctr: controls
AgeMum: maternal age
mBMI: pre-pregnancy Body mass index
GWG: gestational weight gain
PI: ponderal index
MCH: maternal and child health
TC: total cholesterol
LDL-C: low-density lipoprotein–cholesterol
HDL-C: high-density lipoprotein–cholesterol
IRB: Institutional Review Board
BIA: bioelectrical impedance analysis
BMI: Body mass index
zBMIs: BMI z-scores
LCGM: latent class growth model
AIT: akaike Information Criterion
BIC: bayesian Information Criterion
aBIC: adjusted BIC
LMR: Lo-Mendell-Rubin
BLRT: bootstrapped Likelihood Ratio Test
QC: Quality control
MRM: Multiple Reaction Monitoring
RSD: relative standard deviation
SD: standard deviation
BH: Benjamini-Hochberg procedure
PCA: principal component analysis
PC1: first principal component
ELR: early-life risk
MD: metabolic disorder
LASSO: Least Absolute Shrinkage and Selection Operator
ROC: receiver operating characteristic curves
AUC: area under the receiver operating characteristic curve
Low: Low-trajectory group, BMI z values ranging from -0.5 to 0
Middle: Middle-trajectory group, BMI z values ranging from 0 to 0.5
High: High-trajectory group, BMI z values ranging above 0.5
PC: phosphatidylcholine
PE: phophatidylethanolamine
OR: odds ratio
HOMA-IR: Homeostasis Model Assessment-Insulin Resistance.
