# Supplementary material for: Three phosphatidylcholines as a potential early-diagnostic signature for hyperglycemia in preschool-aged children: a cross-sectional pilot study
Source: Front Endocrinol (Lausanne). 2026 Aug 5;17:1878086. doi: 10.3389/fendo.2026.1878086 (PMC13485523; doi:10.3389/fendo.2026.1878086)
Supplement: Supplementary file 1 [file DataSheet1.docx]

Supplementary Material

# 1 Supplementary Data

Supplementary Material should be uploaded separately on submission. Please include any supplementary data, figures and/or tables.

Supplementary material is not typeset so please ensure that all information is clearly presented, the appropriate caption is included in the file and not in the manuscript, and that the style conforms to the rest of the article.

# 2 Supplementary Figures and Tables

## 2.1 Supplementary Figures

**Supplementary Figure 1.** Hypothesized directed acyclic graph (DAG) illustrating the assumed relationships among all study variables.Solid arrows (→) represent directed causal paths hypothesized a priori based on biological plausibility. The dashed line (---) between Lipidomics and Glucose represents an associational link under investigation, without a prespecified causal direction, reflecting the exploratory nature of this study. All variables shown were included as covariates in the multivariable regression models.

B

A

**Supplementary Figure 2.** Principal component analysis of early-life (A) and current metabolic (B) variables.

Glycerophospholipid metabolism

**Supplementary Figure 3.** Pathway analysis of the 36 lipid metabolites.

**Supplementary Figure 4.** Feature selection using LASSO regression. (A) Least Absolute Shrinkage and Selection Operator (LASSO) regression coefficient paths across different λ values. (B) The vertical dashed line indicates the optimal lambda value selected via 10-fold cross-validation. (C) Five lipids retained non-zero coefficients, with the three lipids with the highest absolute coefficient values were defined as signature lipids.

**Supplementary Figure 5.** ROC curves of univariate the three individual lipid markers and their combined diagnostic model. (A-C) Univariate models for PC(P-16:0/18:2) , PC(16:0/20:3), and PC(16:1/18:3), respectively. (D) Combined model of the three lipids.

**Supplementary Figure 6**. Sensitivity Cutoff Comparison of ROC curves between full and clinical model of sensitivity using the original cutoff (FPG ≥ 5.6 mmol/L) and P75 FPG cutoff (FPG ≥ 5.71 mmol/L). (A) ROC curve of full model and base model based on original cutoff. (B) ROC curve of full model and base model based on P75 FPG cutoff. (C) Comparison of AUC between original cutoff and P75 FPG cutoff of three diagnostic model and the sensitivity model. The base model included age and sex. The clinical model included age, sex, BMI, andELR_score. The full model included age, sex, BMI, ELR_score, and three lipids (PC(P-16:0/18:2), PC(16:0/20:3), and PC(16:1/18:3)) as predictors. The sensitivity model included age, sex, BMI, ELR_score, and three lipids (PC(P-16:0/18:2), PC(16:0/20:3), PC(16:1/18:3)), and MD score as predictors

## 2.2 Supplementary Tables

**Supplementary Table 1.** Assessment of Fit Indices for Cohort Latent Class Growth Models.

| number of latent groups | AIC^*^ | BIC^†^ | aBIC^‡^ | Entropy | LMR-P^§^ | BLRT-P^\|\|^ |
| --- | --- | --- | --- | --- | --- | --- |
| 2 | 61151.874 | 61306.778 | 61227.340 | 0.784 | <0.001 | <0.001 |
| 3 | 58711.849 | 58891.538 | 58799.390 | 0.818 | <0.001 | <0.001 |
| 4 | 57814.158 | 58018.632 | 57913.774 | 0.796 | 0.222 | <0.001 |
| 5 | 57271.356 | 57500.614 | 57383.047 | 0.808 | 0.004 | <0.001 |

* AIC: Akaike Information Criterion

† BIC: Bayesian Information Criterion

‡ aBIC: Adjusted Bayesian Information Criterion

§ LMR-p: Lo-Mendell-Rubin p-value

|| BLRT-p: Bootstrapped Likelihood Ratio Test p-value

**Supplementary Table 2.** Early-life risk score and metabolic score of 105samples with PCA*

|  | **Early-life risk score** | **Metabolic score** |
| --- | --- | --- |
| Ctr_M31 | -0.647080328 | -0.445186125 |
| Ctr_M04 | -0.207240388 | 0.435600829 |
| Ctr_M05 | 1.28621103 | -0.031926054 |
| Ctr_M06 | -0.705005683 | 1.933099006 |
| Ctr_M07 | 0.589619071 | 1.175349889 |
| Ctr_M08 | 2.404807238 | -1.236492313 |
| Ctr_M09 | 0.320423657 | 0.766885096 |
| Ctr_M10 | 0.398774196 | 1.101097779 |
| Ctr_M11 | -0.465481618 | -0.026745893 |
| Ctr_M12 | 1.243322263 | 0.113548749 |
| Ctr_M13 | -2.691293363 | -0.802265804 |
| Ctr_M14 | -0.231217058 | 0.133008487 |
| Ctr_M15 | 0.072387639 | -1.012765883 |
| Ctr_M17 | -0.159749861 | -2.717680156 |
| Ctr_M18 | 0.225926751 | -1.704012976 |
| Ctr_M19 | 0.080140164 | -0.945725255 |
| Ctr_M20 | 1.469588233 | 0.919248032 |
| Ctr_M21 | 0.790253612 | 1.168602967 |
| Ctr_M22 | -0.560920675 | 1.208453959 |
| Ctr_M23 | -1.209509698 | 1.687466216 |
| Ctr_M24 | 0.948337498 | 0.267516083 |
| Ctr_M25 | 1.00087693 | 0.668204783 |
| Ctr_M26 | -0.455148922 | -1.348297412 |
| Ctr_M28 | -0.471471086 | -0.383087816 |
| Ctr_M29 | 0.885957298 | 1.673689636 |
| Ctr_M30 | 0.578797116 | -0.095436762 |
| Ctr_Fe01 | -0.006458855 | 0.368586177 |
| Ctr_Fe02 | 2.155996348 | -1.183726561 |
| Ctr_Fe03 | 1.389616786 | -3.040852644 |
| Ctr_Fe04 | -3.654123098 | -1.709715169 |
| Ctr_Fe05 | 1.771921531 | -0.882219634 |
| Ctr_Fe06 | -3.518039093 | 0.285736691 |
| Ctr_Fe07 | 0.706931982 | 1.019879867 |
| Ctr_Fe08 | -0.365732612 | -2.01135397 |
| Ctr_Fe09 | 1.622885492 | 0.391155732 |
| Ctr_Fe10 | 0.610515274 | 0.011314486 |
| Ctr_Fe11 | -1.717239104 | -1.131943046 |
| Ctr_Fe12 | -0.576272759 | -3.139666629 |
| Ctr_Fe13 | 1.767957016 | 0.141035436 |
| Ctr_Fe14 | -0.79061686 | 1.758331032 |
| Ctr_Fe15 | -0.526278021 | 1.371660612 |
| Ctr_Fe16 | 2.1198497 | -2.184554345 |
| Ctr_Fe17 | 2.421719795 | -1.10342714 |
| Ctr_Fe19 | 0.828037527 | -2.721274932 |
| Ctr_Fe21 | -1.229057591 | -3.602255262 |
| Ctr_Fe22 | -1.484044682 | -0.543447204 |
| Ctr_Fe23 | 0.438724815 | -0.375309485 |
| Ctr_Fe24 | -1.872900488 | 2.18362807 |
| Ctr_Fe25 | -1.239468208 | -1.941997615 |
| Ctr_Fe26 | -0.918663775 | 1.118575834 |
| Ctr_Fe27 | 1.446256926 | 0.643303501 |
| Ctr_Fe28 | -0.303879815 | -1.704580712 |
| Ctr_Fe29 | -0.28702329 | -1.283428309 |
| Ctr_Fe30 | -0.449113931 | 0.176396224 |
| HG_M31 | 0.328734236 | -0.818156205 |
| HG_M01 | 1.531808199 | 1.436841586 |
| HG_M03 | -0.48901796 | -2.532923058 |
| HG_M04 | 0.672211605 | 2.471089282 |
| HG_M05 | 1.458353501 | 0.428181651 |
| HG_M06 | 3.037630229 | 1.403258869 |
| HG_M07 | 1.937659925 | -0.505325103 |
| HG_M08 | -1.785774958 | -1.161491339 |
| HG_M09 | -0.473663436 | 1.560217135 |
| HG_M10 | -0.781529824 | -0.782024069 |
| HG_M11 | -2.935855861 | -0.271444302 |
| HG_M12 | 2.529278426 | -1.800294534 |
| HG_M13 | -2.457155544 | 1.033114036 |
| HG_M14 | -2.069117041 | 2.300226206 |
| HG_M15 | -0.387661104 | 0.968955711 |
| HG_M16 | 0.920836169 | 1.207070332 |
| HG_M17 | 1.53456633 | 1.68107479 |
| HG_M18 | 0.063677757 | 0.085118482 |
| HG_M19 | -0.123696339 | 1.794986888 |
| HG_M20 | -1.332516015 | 0.489004009 |
| HG_M21 | 0.006478813 | 0.173175928 |
| HG_M23 | 0.532328559 | 2.040995632 |
| HG_M24 | -1.320574839 | -0.080976554 |
| HG_M25 | -1.904510992 | 1.831190361 |
| HG_M26 | -0.183173579 | -1.415186554 |
| HG_M27 | -0.489008993 | 0.534615529 |
| HG_M29 | 2.040041016 | 0.222352124 |
| HG_M30 | -2.125428973 | 0.607926664 |
| HG_Fe02 | -0.571753031 | -0.364213924 |
| HG_Fe03 | 0.076172886 | 1.092689204 |
| HG_Fe05 | -2.064407904 | -2.021977286 |
| HG_Fe06 | 0.325672623 | -0.234156632 |
| HG_Fe08 | 0.824091638 | 0.162207564 |
| HG_Fe09 | -0.503968119 | 0.428240544 |
| HG_Fe12 | -0.585812273 | 1.477978796 |
| HG_Fe13 | -1.002946445 | -0.321937045 |
| HG_Fe14 | 0.989898663 | 0.036837838 |
| HG_Fe15 | 0.196963106 | -1.630800071 |
| HG_Fe16 | 0.262545147 | 0.693748866 |
| HG_Fe17 | 0.290421132 | -1.925434465 |
| HG_Fe18 | 1.662148756 | 1.090188321 |
| HG_Fe20 | -3.170653255 | -0.926550319 |
| HG_Fe21 | 1.370522035 | 1.762321414 |
| HG_Fe22 | -0.796089269 | -0.424260826 |
| HG_Fe23 | 1.903211918 | 2.826272078 |
| HG_Fe24 | -0.406576575 | -0.861384579 |
| HG_Fe25 | 1.081757206 | -0.851116343 |
| HG_Fe27 | -2.200211614 | 1.205757194 |
| HG_Fe28 | 0.777525191 | 2.577673369 |
| HG_Fe29 | 1.615917411 | -0.954328243 |
| HG_Fe30 | -0.642153561 | 0.84867098 |

* M: male; Fe: female

**Supplementary Table 3.** The contribution and loadings of early-life risk variables in PC1

|  | Contributions | Loadings |
| --- | --- | --- |
| income | 2.378990483 | -0.212948219 |
| mBMI_zscore | 0.266359124 | 0.071254361 |
| logGWG^*^ | 4.290791591 | 0.285987052 |
| breastfeeding | 16.55552614 | 0.561757856 |
| z_GW^†^ | 27.07959188 | 0.718453588 |
| z_AgeMum^‡^ | 13.70431213 | -0.511100471 |
| 0-2 year Growth tragectory | 7.398682425 | 0.375538834 |
| logPI^§^ | 28.32574624 | 0.734798635 |

* logGWG: log2 transformed of gestational weight gain

† z_GW: z_score of gestational age

‡ z_ AgeMum: z_score of mother's age when gave birth to baby

§ logPI: log2 transformed of ponderal index

**Supplementary Table 4.** The contributions of metabolic disorder variables in PCA

|  | **Contributions** | **Loadings** |
| --- | --- | --- |
| TC | 43.17019767 | 0.909257871 |
| HDL | 0.106082502 | -0.045073048 |
| LDL | 45.9562861 | 0.938139696 |
| TG | 10.58421889 | 0.450219661 |
| UA | 0.183214835 | 0.059234587 |

TG: triglyceride;

TC: total cholesterol;

LDL-C: low-density lipoprotein–cholesterol;

HDL-C: high-density lipoprotein–cholesterol;

UA: uric acid;

**Supplementary** Table 5. Linear regression analysis of FBG and 376 lipids. pFDR threshold of less than 0.2.

|  | ***β*** | **CI_lower** | **CI_upper** | **Pr(>\|t\|)** | **bonferroni** | **BH** | **log(FC)** |
| --- | --- | --- | --- | --- | --- | --- | --- |
| **CE(16:0)** | **0.23** | **0.05** | **0.40** | **0.0136** | 1 | **0.14518** | 0.1965 |
| CE(16:1) | 0.08 | -0.10 | 0.26 | 0.367 | 1 | 0.67976 | 0.1151 |
| CE(17:0) | 0.15 | -0.03 | 0.32 | 0.114 | 1 | 0.44650 | 0.1094 |
| CE(18:0) | -0.20 | -0.38 | -0.02 | 0.0314 | 1 | 0.24095 | -0.1315 |
| CE(18:2) | -0.14 | -0.32 | 0.04 | 0.13 | 1 | 0.47456 | -0.0566 |
| CE(20:0) | 0.06 | -0.12 | 0.24 | 0.49 | 1 | 0.75974 | 0.0989 |
| CE(20:2) | 0.01 | -0.17 | 0.19 | 0.915 | 1 | 0.96042 | 0.1046 |
| Cer(d18:1/16:0) | 0.02 | -0.16 | 0.20 | 0.801 | 1 | 0.93040 | -0.0197 |
| Cer(d18:1/16:1) | -0.14 | -0.32 | 0.04 | 0.125 | 1 | 0.46907 | -0.1089 |
| Cer(d18:1/17:0) | 0.03 | -0.15 | 0.22 | 0.711 | 1 | 0.90593 | 0.0276 |
| Cer(d18:1/18:0) | 0.09 | -0.09 | 0.27 | 0.354 | 1 | 0.67224 | 0.0410 |
| Cer(d18:1/18:1) | -0.06 | -0.24 | 0.12 | 0.514 | 1 | 0.76976 | -0.0698 |
| Cer(d18:1/18:4) | -0.13 | -0.31 | 0.05 | 0.161 | 1 | 0.51302 | -0.0827 |
| Cer(d18:1/20:0) | 0.04 | -0.14 | 0.22 | 0.663 | 1 | 0.87733 | -0.0123 |
| Cer(d18:1/20:1) | 0.05 | -0.13 | 0.23 | 0.607 | 1 | 0.83739 | 0.0086 |
| Cer(d18:1/20:2) | 0.02 | -0.16 | 0.20 | 0.818 | 1 | 0.93040 | -0.0027 |
| Cer(d18:1/20:3) | 0.03 | -0.15 | 0.21 | 0.744 | 1 | 0.91725 | -0.0098 |
| Cer(d18:1/20:4) | -0.03 | -0.21 | 0.15 | 0.758 | 1 | 0.91725 | -0.0317 |
| Cer(d18:1/20:5) | -0.11 | -0.29 | 0.07 | 0.232 | 1 | 0.58626 | -0.0785 |
| Cer(d18:1/22:0) | 0.01 | -0.17 | 0.19 | 0.944 | 1 | 0.97925 | -0.0114 |
| Cer(d18:1/22:1) | -0.02 | -0.20 | 0.16 | 0.808 | 1 | 0.93040 | -0.0601 |
| Cer(d18:1/22:2) | -0.04 | -0.22 | 0.14 | 0.643 | 1 | 0.86440 | -0.0805 |
| Cer(d18:1/22:3) | -0.04 | -0.22 | 0.14 | 0.66 | 1 | 0.87733 | -0.0537 |
| Cer(d18:1/22:4) | -0.03 | -0.21 | 0.16 | 0.782 | 1 | 0.92463 | -0.0448 |
| Cer(d18:1/22:5) | -0.08 | -0.26 | 0.10 | 0.378 | 1 | 0.69671 | -0.0682 |
| Cer(d18:1/22:6) | 0.06 | -0.12 | 0.24 | 0.52 | 1 | 0.76976 | 0.0242 |
| DG(12:0/18:2) | 0.01 | -0.17 | 0.19 | 0.884 | 1 | 0.96042 | -0.1326 |
| DG(12:0/18:3) | 0.01 | -0.17 | 0.19 | 0.889 | 1 | 0.96042 | -0.0856 |
| DG(14:0/16:0) | -0.12 | -0.30 | 0.06 | 0.193 | 1 | 0.56111 | -0.0090 |
| **DG(14:0/18:0)** | **-0.25** | **-0.43** | **-0.08** | **0.00558** | 1 | **0.08392** | -0.2027 |
| **DG(14:0/18:1)** | **0.23** | **0.05** | **0.40** | **0.0134** | 1 | **0.14518** | 0.1546 |
| DG(14:0/18:2) | 0.03 | -0.15 | 0.21 | 0.763 | 1 | 0.91725 | -0.0014 |
| DG(14:1/18:1) | -0.12 | -0.30 | 0.06 | 0.203 | 1 | 0.56961 | -0.2095 |
| DG(14:1/22:4) | -0.08 | -0.26 | 0.10 | 0.387 | 1 | 0.70637 | -0.1754 |
| DG(14:1/22:5) | -0.13 | -0.31 | 0.05 | 0.15 | 1 | 0.48621 | -0.1908 |
| DG(14:1/22:6) | -0.04 | -0.22 | 0.14 | 0.685 | 1 | 0.89121 | -0.1128 |
| DG(16:0/16:0) | -0.07 | -0.25 | 0.11 | 0.428 | 1 | 0.72121 | -0.0105 |
| DG(16:0/16:1) | 0.01 | -0.18 | 0.19 | 0.948 | 1 | 0.97925 | -0.1093 |
| **DG(16:0/18:0)** | **-0.25** | **-0.42** | **-0.07** | **0.0063** | 1 | **0.08460** | -0.0904 |
| DG(16:0/18:1) | -0.13 | -0.30 | 0.05 | 0.175 | 1 | 0.54243 | -0.1292 |
| DG(16:0/18:2) | -0.17 | -0.35 | 0.01 | 0.0613 | 1 | 0.31147 | -0.1237 |
| DG(16:0/20:4) | -0.09 | -0.27 | 0.09 | 0.31 | 1 | 0.62667 | -0.1909 |
| DG(16:1/16:1) | -0.08 | -0.26 | 0.10 | 0.397 | 1 | 0.71422 | -0.0766 |
| DG(16:1/18:0) | -0.19 | -0.36 | -0.01 | 0.0437 | 1 | 0.26998 | -0.4620 |
| DG(16:1/18:1) | -0.01 | -0.19 | 0.17 | 0.915 | 1 | 0.96042 | 0.0757 |
| DG(16:1/18:2) | 0.06 | -0.12 | 0.24 | 0.505 | 1 | 0.76976 | 0.2449 |
| DG(18:0/18:0) | -0.03 | -0.22 | 0.15 | 0.707 | 1 | 0.90419 | 0.1186 |
| **DG(18:0/18:1)** | **-0.22** | **-0.39** | **-0.04** | **0.0177** | 1 | **0.16114** | -0.2519 |
| DG(18:0/18:2) | 0.11 | -0.07 | 0.29 | 0.232 | 1 | 0.58626 | 0.1554 |
| **DG(18:0/20:0)** | **0.26** | **0.08** | **0.43** | **0.00457** | 1 | **0.07471** | 0.3894 |
| DG(18:1/18:1) | -0.06 | -0.24 | 0.12 | 0.535 | 1 | 0.77369 | -0.0289 |
| DG(18:1/18:2) | -0.16 | -0.34 | 0.02 | 0.0837 | 1 | 0.37466 | -0.1064 |
| DG(18:1/18:3) | 0.18 | 0.00 | 0.36 | 0.0505 | 1 | 0.27924 | 0.1801 |
| DG(18:1/20:1) | -0.10 | -0.28 | 0.08 | 0.29 | 1 | 0.62667 | -0.1836 |
| DG(18:2/18:2) | -0.15 | -0.33 | 0.03 | 0.111 | 1 | 0.43933 | -0.1240 |
| DG(20:0/22:1) | 0.07 | -0.11 | 0.25 | 0.44 | 1 | 0.72121 | 0.0344 |
| **DG(20:0/22:6)** | **-0.30** | **-0.47** | **-0.13** | **0.000852** | 0.320352 | **0.03534** | -0.2728 |
| HexCer(d18:1/12:0) | 0.10 | -0.09 | 0.28 | 0.304 | 1 | 0.62667 | 0.0182 |
| HexCer(d18:1/14:0) | 0.07 | -0.11 | 0.25 | 0.436 | 1 | 0.72121 | -0.0263 |
| HexCer(d18:1/16:0) | -0.03 | -0.21 | 0.15 | 0.742 | 1 | 0.91725 | -0.0895 |
| HexCer(d18:1/16:1) | -0.10 | -0.28 | 0.08 | 0.298 | 1 | 0.62667 | -0.0717 |
| HexCer(d18:1/18:0) | -0.11 | -0.29 | 0.07 | 0.239 | 1 | 0.58735 | -0.0742 |
| HexCer(d18:1/18:1) | -0.08 | -0.26 | 0.10 | 0.364 | 1 | 0.67754 | -0.0866 |
| HexCer(d18:1/18:2) | -0.04 | -0.22 | 0.14 | 0.694 | 1 | 0.89671 | -0.0439 |
| HexCer(d18:1/20:0) | -0.09 | -0.27 | 0.09 | 0.327 | 1 | 0.64373 | -0.0643 |
| HexCer(d18:1/20:1) | 0.02 | -0.16 | 0.20 | 0.84 | 1 | 0.93721 | 0.0486 |
| HexCer(d18:1/20:2) | -0.01 | -0.19 | 0.17 | 0.911 | 1 | 0.96042 | -0.0527 |
| HexCer(d18:1/22:0) | 0.06 | -0.12 | 0.24 | 0.508 | 1 | 0.76976 | 0.0079 |
| HexCer(d18:1/22:1) | -0.12 | -0.30 | 0.06 | 0.188 | 1 | 0.55660 | -0.1151 |
| HexCer(d18:1/22:2) | 0.02 | -0.16 | 0.20 | 0.848 | 1 | 0.94055 | -0.0474 |
| HexCer(d18:1/22:3) | 0.00 | -0.18 | 0.18 | 0.971 | 1 | 0.98587 | -0.0515 |
| HexCer(d18:1/22:6) | 0.07 | -0.11 | 0.25 | 0.433 | 1 | 0.72121 | 0.0265 |
| LPC(12:0) | 0.15 | -0.03 | 0.33 | 0.105 | 1 | 0.42913 | 0.2396 |
| LPC(14:1) | 0.08 | -0.10 | 0.26 | 0.393 | 1 | 0.71042 | -0.0071 |
| LPC(15:0) | 0.02 | -0.16 | 0.20 | 0.824 | 1 | 0.93040 | -0.0198 |
| LPC(16:0) | 0.01 | -0.17 | 0.20 | 0.875 | 1 | 0.95471 | -0.0071 |
| LPC(16:1) | 0.08 | -0.10 | 0.26 | 0.406 | 1 | 0.72008 | 0.0407 |
| LPC(18:0) | -0.03 | -0.21 | 0.16 | 0.78 | 1 | 0.92463 | -0.0126 |
| LPC(18:1) | -0.14 | -0.32 | 0.04 | 0.141 | 1 | 0.47762 | -0.0497 |
| LPC(18:2) | -0.01 | -0.19 | 0.17 | 0.913 | 1 | 0.96042 | 0.0010 |
| LPC(18:3) | 0.08 | -0.11 | 0.26 | 0.414 | 1 | 0.72067 | 0.0560 |
| LPC(20:0) | -0.02 | -0.20 | 0.17 | 0.867 | 1 | 0.95041 | 0.0174 |
| LPC(20:3) | 0.12 | -0.06 | 0.30 | 0.211 | 1 | 0.57067 | 0.0632 |
| LPC(20:4) | 0.06 | -0.12 | 0.24 | 0.508 | 1 | 0.76976 | 0.0137 |
| LPC(20:5) | -0.07 | -0.25 | 0.11 | 0.456 | 1 | 0.73138 | -0.1108 |
| LPC(22:1) | 0.19 | 0.01 | 0.37 | 0.0398 | 1 | 0.26991 | 0.1827 |
| LPC(22:4) | -0.03 | -0.21 | 0.15 | 0.718 | 1 | 0.90593 | -0.0332 |
| LPC(22:6) | -0.12 | -0.30 | 0.06 | 0.207 | 1 | 0.57067 | -0.0826 |
| LPC(24:0) | -0.07 | -0.25 | 0.11 | 0.445 | 1 | 0.72121 | -0.0596 |
| LPC(O-16:0) | 0.05 | -0.13 | 0.23 | 0.593 | 1 | 0.82859 | -0.0057 |
| LPC(O-18:0) | -0.08 | -0.26 | 0.10 | 0.41 | 1 | 0.72037 | -0.0610 |
| LPC(P-18:1) | 0.07 | -0.11 | 0.25 | 0.458 | 1 | 0.73138 | -0.0691 |
| LPC(P-20:0) | -0.12 | -0.30 | 0.06 | 0.198 | 1 | 0.56685 | -0.0787 |
| LPE(16:0) | 0.05 | -0.13 | 0.23 | 0.595 | 1 | 0.82859 | 0.0340 |
| LPE(16:1) | 0.14 | -0.04 | 0.32 | 0.12 | 1 | 0.46335 | 0.1566 |
| LPE(18:0) | 0.12 | -0.06 | 0.30 | 0.202 | 1 | 0.56961 | 0.0797 |
| LPE(18:1) | -0.12 | -0.30 | 0.06 | 0.194 | 1 | 0.56111 | -0.1018 |
| LPE(20:0) | -0.18 | -0.36 | 0.00 | 0.0484 | 1 | 0.27573 | -0.0963 |
| LPE(20:1) | -0.08 | -0.27 | 0.10 | 0.36 | 1 | 0.67717 | -0.0135 |
| LPE(20:2) | 0.04 | -0.14 | 0.22 | 0.64 | 1 | 0.86440 | 0.0415 |
| LPE(20:3) | 0.08 | -0.10 | 0.26 | 0.387 | 1 | 0.70637 | 0.1128 |
| LPE(20:4) | 0.03 | -0.15 | 0.21 | 0.718 | 1 | 0.90593 | 0.0286 |
| LPE(20:5) | -0.07 | -0.25 | 0.11 | 0.434 | 1 | 0.72121 | -0.0668 |
| LPE(22:0) | 0.04 | -0.14 | 0.22 | 0.688 | 1 | 0.89203 | 0.0389 |
| LPE(22:4) | 0.02 | -0.16 | 0.20 | 0.805 | 1 | 0.93040 | 0.0002 |
| LPE(22:5) | 0.08 | -0.10 | 0.26 | 0.406 | 1 | 0.72008 | 0.1127 |
| LPE(22:6) | -0.10 | -0.28 | 0.08 | 0.268 | 1 | 0.62667 | -0.0275 |
| **MG(16:0)** | **-0.25** | **-0.43** | **-0.08** | **0.00594** | 1 | **0.08460** | -0.2414 |
| MG(16:1) | 0.04 | -0.15 | 0.22 | 0.701 | 1 | 0.90266 | 0.0043 |
| MG(18:0) | -0.07 | -0.25 | 0.11 | 0.471 | 1 | 0.73790 | -0.1253 |
| MG(18:1) | 0.05 | -0.13 | 0.23 | 0.578 | 1 | 0.81702 | -0.0383 |
| **MG(18:2)** | **0.27** | **0.09** | **0.44** | **0.00342** | 1 | **0.07248** | 0.2872 |
| **MG(22:5)** | **-0.30** | **-0.47** | **-0.13** | **0.00094** | 0.35344 | **0.03534** | -0.1612 |
| PC(O-16:0/16:0) | 0.21 | 0.03 | 0.38 | 0.025 | 1 | 0.20435 | 0.0799 |
| PC(O-16:0/16:1) | -0.05 | -0.23 | 0.13 | 0.592 | 1 | 0.82859 | -0.0195 |
| PC(O-16:0/18:0) | 0.02 | -0.16 | 0.20 | 0.813 | 1 | 0.93040 | 0.0079 |
| PC(O-16:0/18:1) | 0.09 | -0.09 | 0.27 | 0.322 | 1 | 0.63920 | -0.0103 |
| PC(O-16:0/18:2) | -0.04 | -0.22 | 0.14 | 0.637 | 1 | 0.86440 | -0.0419 |
| **PC(O-16:0/18:3)** | **0.29** | **0.12** | **0.46** | **0.00144** | 0.54144 | **0.04165** | 0.1310 |
| PC(O-16:0/20:1) | 0.19 | 0.01 | 0.36 | 0.0427 | 1 | 0.26998 | 0.0549 |
| PC(O-16:0/20:2) | 0.06 | -0.12 | 0.24 | 0.523 | 1 | 0.77117 | -0.0234 |
| PC(O-16:0/20:4) | 0.03 | -0.15 | 0.21 | 0.742 | 1 | 0.91725 | -0.0044 |
| PC(O-16:0/22:5) | -0.01 | -0.19 | 0.17 | 0.911 | 1 | 0.96042 | -0.0452 |
| PC(O-16:0/22:6) | 0.19 | 0.01 | 0.37 | 0.0366 | 1 | 0.25965 | 0.0613 |
| PC(P-16:0/16:0) | 0.10 | -0.08 | 0.28 | 0.297 | 1 | 0.62667 | 0.0638 |
| PC(P-16:0/18:0) | 0.05 | -0.13 | 0.23 | 0.586 | 1 | 0.82523 | -0.0141 |
| PC(P-16:0/18:1) | 0.04 | -0.14 | 0.22 | 0.669 | 1 | 0.87952 | 0.0003 |
| **PC(P-16:0/18:2)** | **0.29** | **0.12** | **0.46** | **0.00134** | 0.50384 | **0.04165** | 0.2239 |
| PC(P-16:0/20:0) | 0.15 | -0.02 | 0.33 | 0.0936 | 1 | 0.40505 | 0.0599 |
| PC(P-16:0/20:1) | 0.07 | -0.11 | 0.25 | 0.429 | 1 | 0.72121 | -0.0059 |
| PC(P-16:0/20:2) | 0.10 | -0.08 | 0.28 | 0.292 | 1 | 0.62667 | 0.0057 |
| PC(P-16:0/20:4) | -0.06 | -0.24 | 0.12 | 0.497 | 1 | 0.76274 | -0.1716 |
| PC(P-16:0/22:6) | 0.03 | -0.15 | 0.21 | 0.741 | 1 | 0.91725 | 0.0234 |
| PC(O-18:0/16:0) | 0.09 | -0.09 | 0.28 | 0.305 | 1 | 0.62667 | 0.0303 |
| PC(O-18:0/16:1) | 0.08 | -0.10 | 0.26 | 0.406 | 1 | 0.72008 | -0.0110 |
| PC(O-18:0/18:0) | 0.07 | -0.11 | 0.25 | 0.441 | 1 | 0.72121 | 0.0338 |
| PC(O-18:0/18:1) | 0.10 | -0.08 | 0.28 | 0.284 | 1 | 0.62667 | 0.0939 |
| PC(O-18:0/18:2) | 0.10 | -0.08 | 0.28 | 0.272 | 1 | 0.62667 | 0.0002 |
| PC(O-18:0/18:3) | 0.04 | -0.14 | 0.22 | 0.661 | 1 | 0.87733 | -0.0258 |
| PC(O-18:0/20:4) | 0.11 | -0.07 | 0.29 | 0.225 | 1 | 0.58345 | 0.0137 |
| PC(P-18:0/14:0) | -0.01 | -0.19 | 0.17 | 0.9 | 1 | 0.96042 | -0.0210 |
| PC(P-18:0/16:0) | 0.02 | -0.16 | 0.20 | 0.798 | 1 | 0.93040 | -0.0370 |
| PC(P-18:0/16:1) | -0.02 | -0.20 | 0.16 | 0.824 | 1 | 0.93040 | -0.0320 |
| PC(P-18:0/18:0) | 0.01 | -0.17 | 0.20 | 0.876 | 1 | 0.95471 | 0.0180 |
| PC(P-18:0/18:1) | 0.06 | -0.12 | 0.24 | 0.516 | 1 | 0.76976 | -0.0089 |
| PC(P-18:0/18:2) | 0.09 | -0.09 | 0.27 | 0.336 | 1 | 0.64981 | 0.0013 |
| PC(P-18:0/18:3) | 0.13 | -0.05 | 0.31 | 0.146 | 1 | 0.48581 | 0.0426 |
| PC(O-18:1/16:0) | 0.11 | -0.07 | 0.29 | 0.214 | 1 | 0.57067 | 0.0167 |
| PC(O-18:1/16:1) | -0.03 | -0.21 | 0.15 | 0.763 | 1 | 0.91725 | -0.0364 |
| PC(O-18:1/18:0) | 0.12 | -0.06 | 0.30 | 0.188 | 1 | 0.55660 | 0.0485 |
| PC(O-18:1/18:1) | 0.05 | -0.13 | 0.23 | 0.562 | 1 | 0.79740 | 0.0001 |
| PC(O-18:1/18:2) | 0.11 | -0.07 | 0.29 | 0.237 | 1 | 0.58626 | 0.0105 |
| PC(O-18:1/18:3) | 0.15 | -0.03 | 0.33 | 0.111 | 1 | 0.43933 | 0.0249 |
| PC(O-18:1/20:4) | 0.04 | -0.14 | 0.22 | 0.645 | 1 | 0.86440 | -0.0098 |
| PC(O-18:2/16:0) | -0.05 | -0.23 | 0.14 | 0.62 | 1 | 0.85080 | -0.0424 |
| **PC(O-18:2/16:1)** | **0.23** | **0.05** | **0.40** | **0.0134** | 1 | **0.14518** | 0.1178 |
| PC(O-18:2/18:0) | 0.13 | -0.05 | 0.31 | 0.17 | 1 | 0.53714 | 0.0236 |
| PC(O-18:2/18:1) | 0.10 | -0.08 | 0.28 | 0.294 | 1 | 0.62667 | 0.0216 |
| PC(O-18:2/18:2) | 0.09 | -0.09 | 0.27 | 0.323 | 1 | 0.63920 | 0.0179 |
| PC(O-18:2/18:3) | -0.04 | -0.22 | 0.14 | 0.665 | 1 | 0.87733 | -0.1304 |
| PC(P-18:2/18:0) | 0.09 | -0.09 | 0.27 | 0.337 | 1 | 0.64981 | 0.0131 |
| PC(P-18:2/18:1) | 0.01 | -0.18 | 0.19 | 0.948 | 1 | 0.97925 | -0.0427 |
| PC(P-18:2/18:2) | -0.02 | -0.21 | 0.16 | 0.792 | 1 | 0.93040 | -0.0970 |
| PC(P-18:2/20:3) | 0.14 | -0.04 | 0.32 | 0.137 | 1 | 0.47762 | 0.0464 |
| PC(P-18:2/20:4) | 0.07 | -0.11 | 0.25 | 0.458 | 1 | 0.73138 | 0.0542 |
| PC(P-18:2/20:5) | 0.07 | -0.11 | 0.26 | 0.42 | 1 | 0.72121 | 0.0303 |
| **SM(d18:1/12:0** | **0.27** | **0.10** | **0.45** | **0.00273** | 1 | **0.06439** | 0.2275 |
| SM(d18:1/14:0) | 0.07 | -0.11 | 0.25 | 0.47 | 1 | 0.73790 | 0.0026 |
| **SM(d18:1/14:1)** | **0.29** | **0.11** | **0.46** | **0.00163** | 0.61288 | **0.04378** | 0.1097 |
| **SM(d18:1/16:0)** | **0.21** | **0.04** | **0.39** | **0.0196** | 1 | **0.16749** | 0.0684 |
| **SM(d18:1/16:1)** | **0.39** | **0.23** | **0.56** | **0.00000889** | 0.00334264 | **0.00167** | 0.2308 |
| **SM(d18:1/17:0)** | **0.24** | **0.07** | **0.42** | **0.0075** | 1 | **0.09097** | 0.2356 |
| **SM(d18:1/18:0)** | **0.26** | **0.08** | **0.43** | **0.00457** | 1 | **0.07471** | 0.1919 |
| **SM(d18:1/18:1)** | **0.35** | **0.18** | **0.52** | **0.0000994** | 0.0373744 | **0.00934** | 0.3071 |
| SM(d18:1/18:2) | 0.10 | -0.08 | 0.28 | 0.298 | 1 | 0.62667 | 0.0425 |
| SM(d18:1/18:3) | 0.20 | 0.02 | 0.38 | 0.0307 | 1 | 0.24048 | 0.1325 |
| SM(d18:1/18:4) | -0.07 | -0.25 | 0.11 | 0.433 | 1 | 0.72121 | -0.0284 |
| SM(d18:1/20:0) | -0.01 | -0.19 | 0.17 | 0.914 | 1 | 0.96042 | -0.0278 |
| SM(d18:1/20:1) | -0.09 | -0.27 | 0.09 | 0.335 | 1 | 0.64981 | -0.0693 |
| SM(d18:1/20:2) | -0.04 | -0.22 | 0.14 | 0.645 | 1 | 0.86440 | -0.0811 |
| SM(d18:1/20:3) | 0.03 | -0.15 | 0.21 | 0.758 | 1 | 0.91725 | -0.0361 |
| **SM(d18:1/20:4)** | **0.22** | **0.04** | **0.40** | **0.0168** | 1 | **0.16114** | 0.3511 |
| SM(d18:1/20:5) | -0.11 | -0.29 | 0.07 | 0.227 | 1 | 0.58460 | -0.1022 |
| SM(d18:1/22:0) | 0.00 | -0.18 | 0.18 | 0.975 | 1 | 0.98587 | 0.0211 |
| SM(d18:1/22:1) | -0.02 | -0.21 | 0.16 | 0.795 | 1 | 0.93040 | -0.0578 |
| SM(d18:1/22:2) | -0.06 | -0.24 | 0.12 | 0.491 | 1 | 0.75974 | -0.0530 |
| SM(d18:1/22:3) | 0.10 | -0.08 | 0.28 | 0.281 | 1 | 0.62667 | 0.0336 |
| SM(d18:1/22:4) | -0.02 | -0.20 | 0.16 | 0.857 | 1 | 0.94550 | -0.0733 |
| SM(d18:1/22:5) | -0.09 | -0.27 | 0.09 | 0.332 | 1 | 0.64981 | -0.0691 |
| SM(d18:1/22:6) | -0.11 | -0.29 | 0.07 | 0.233 | 1 | 0.58626 | -0.0708 |
| Sphingosine | 0.11 | -0.07 | 0.29 | 0.218 | 1 | 0.57320 | 0.1057 |
| TG(46:2) | 0.18 | 0.01 | 0.36 | 0.0459 | 1 | 0.27394 | 0.1820 |
| TG(48:0) | 0.16 | -0.02 | 0.34 | 0.0806 | 1 | 0.36958 | 0.3064 |
| TG(48:1) | 0.17 | -0.01 | 0.35 | 0.0612 | 1 | 0.31147 | 0.3644 |
| TG(48:2) | 0.20 | 0.02 | 0.38 | 0.0306 | 1 | 0.24048 | 0.3953 |
| TG(50:0) | 0.00 | -0.19 | 0.18 | 0.96 | 1 | 0.98354 | 0.0353 |
| TG(50:1) | 0.07 | -0.11 | 0.25 | 0.443 | 1 | 0.72121 | 0.0799 |
| TG(50:2) | 0.08 | -0.10 | 0.26 | 0.362 | 1 | 0.67717 | 0.1933 |
| TG(50:3) | 0.11 | -0.07 | 0.29 | 0.214 | 1 | 0.57067 | 0.0899 |
| **TG(50:4)** | **0.25** | **0.07** | **0.42** | **0.00626** | 1 | **0.08460** | 0.2393 |
| TG(52:2) | -0.03 | -0.21 | 0.15 | 0.777 | 1 | 0.92453 | 0.0068 |
| TG(52:3) | -0.06 | -0.24 | 0.12 | 0.497 | 1 | 0.76274 | 0.0152 |
| TG(52:4) | 0.05 | -0.13 | 0.23 | 0.599 | 1 | 0.83108 | 0.0986 |
| TG(52:5) | 0.10 | -0.08 | 0.28 | 0.292 | 1 | 0.62667 | 0.1180 |
| TG(52:6) | -0.03 | -0.21 | 0.15 | 0.76 | 1 | 0.91725 | 0.0384 |
| TG(54:1) | -0.04 | -0.22 | 0.14 | 0.637 | 1 | 0.86440 | -0.0052 |
| TG(54:2) | -0.14 | -0.32 | 0.04 | 0.126 | 1 | 0.46907 | -0.0654 |
| TG(54:3) | -0.14 | -0.32 | 0.04 | 0.121 | 1 | 0.46335 | -0.0509 |
| TG(54:4) | -0.10 | -0.28 | 0.08 | 0.297 | 1 | 0.62667 | -0.0225 |
| TG(54:5) | -0.07 | -0.25 | 0.11 | 0.43 | 1 | 0.72121 | -0.0403 |
| TG(54:6) | -0.02 | -0.20 | 0.16 | 0.86 | 1 | 0.94550 | 0.0515 |
| FA(12:0) | 0.07 | -0.11 | 0.25 | 0.444 | 1 | 0.72121 | 0.0272 |
| FA(12:1) | -0.10 | -0.28 | 0.08 | 0.265 | 1 | 0.62667 | 0.0420 |
| FA(14:0) | 0.06 | -0.12 | 0.24 | 0.53 | 1 | 0.77240 | 0.0142 |
| FA(14:1) | -0.10 | -0.28 | 0.08 | 0.263 | 1 | 0.62667 | 0.0117 |
| FA(16:0) | -0.09 | -0.27 | 0.09 | 0.31 | 1 | 0.62667 | -0.0449 |
| **FA(16:1)** | **-0.21** | **-0.39** | **-0.03** | **0.0228** | 1 | **0.19051** | -0.0589 |
| FA(16:2) | -0.04 | -0.22 | 0.14 | 0.646 | 1 | 0.86440 | 0.0343 |
| FA(17:0) | 0.02 | -0.16 | 0.20 | 0.814 | 1 | 0.93040 | -0.0681 |
| **FA(17:1)** | **-0.33** | **-0.50** | **-0.16** | **0.00021** | 0.07896 | **0.01579** | -0.1867 |
| FA(18:0) | -0.19 | -0.37 | -0.02 | 0.034 | 1 | 0.24585 | -0.0769 |
| FA(18:1) | 0.15 | -0.03 | 0.33 | 0.0981 | 1 | 0.40984 | 0.0147 |
| FA(18:2) | -0.08 | -0.26 | 0.10 | 0.39 | 1 | 0.70841 | -0.0409 |
| FA(18:3) | -0.16 | -0.34 | 0.01 | 0.0739 | 1 | 0.34722 | -0.0253 |
| FA(18:4) | 0.06 | -0.13 | 0.24 | 0.55 | 1 | 0.78931 | 0.1284 |
| FA(20:0) | 0.18 | 0.00 | 0.36 | 0.0535 | 1 | 0.28952 | 0.0908 |
| FA(20:1) | -0.17 | -0.35 | 0.01 | 0.0624 | 1 | 0.31283 | -0.0415 |
| FA(20:2) | -0.02 | -0.20 | 0.16 | 0.86 | 1 | 0.94550 | 0.0354 |
| FA(20:3) | 0.17 | -0.01 | 0.35 | 0.0668 | 1 | 0.32619 | 0.1079 |
| FA(20:4) | 0.02 | -0.16 | 0.20 | 0.839 | 1 | 0.93721 | 0.0064 |
| FA(20:5) | 0.14 | -0.04 | 0.32 | 0.122 | 1 | 0.46335 | 0.1116 |
| FA(22:0) | 0.02 | -0.16 | 0.20 | 0.83 | 1 | 0.93437 | -0.1116 |
| FA(22:1) | -0.04 | -0.22 | 0.14 | 0.675 | 1 | 0.88432 | -0.0050 |
| FA(22:2) | 0.09 | -0.09 | 0.27 | 0.318 | 1 | 0.63600 | 0.0961 |
| FA(22:3) | 0.10 | -0.08 | 0.28 | 0.278 | 1 | 0.62667 | 0.1014 |
| FA(22:4) | -0.01 | -0.19 | 0.17 | 0.927 | 1 | 0.96552 | 0.0379 |
| FA(22:5) | -0.15 | -0.33 | 0.03 | 0.109 | 1 | 0.43933 | -0.0640 |
| FA(22:6) | -0.13 | -0.31 | 0.05 | 0.15 | 1 | 0.48621 | -0.0849 |
| LPA(16:0) | 0.02 | -0.16 | 0.20 | 0.8 | 1 | 0.93040 | 0.0025 |
| LPA(18:0) | -0.12 | -0.30 | 0.06 | 0.185 | 1 | 0.55660 | -0.0714 |
| LPA(18:1) | 0.03 | -0.15 | 0.21 | 0.775 | 1 | 0.92453 | -0.0530 |
| LPA(18:2) | -0.12 | -0.30 | 0.06 | 0.181 | 1 | 0.55330 | -0.1272 |
| PC(12:0/16:0) | 0.09 | -0.09 | 0.27 | 0.308 | 1 | 0.62667 | -0.0361 |
| PC(14:0/16:0) | 0.18 | 0.00 | 0.36 | 0.0483 | 1 | 0.27573 | 0.0939 |
| **PC(14:0/16:1)** | **0.27** | **0.10** | **0.45** | **0.00274** | 1 | **0.06439** | 0.3986 |
| PC(14:1/16:0) | -0.07 | -0.25 | 0.11 | 0.461 | 1 | 0.73138 | -0.1481 |
| PC(14:1/16:1) | 0.11 | -0.07 | 0.29 | 0.234 | 1 | 0.58626 | 0.0111 |
| PC(16:0/16:0) | 0.07 | -0.11 | 0.25 | 0.431 | 1 | 0.72121 | 0.0118 |
| PC(16:0/16:1) | 0.16 | -0.02 | 0.34 | 0.0827 | 1 | 0.37464 | 0.1638 |
| PC(16:0/18:0) | 0.15 | -0.03 | 0.33 | 0.0948 | 1 | 0.40505 | 0.0808 |
| PC(16:0/18:1) | 0.19 | 0.01 | 0.36 | 0.0436 | 1 | 0.26998 | 0.0791 |
| PC(16:0/18:2) | -0.13 | -0.31 | 0.05 | 0.174 | 1 | 0.54243 | -0.0760 |
| PC(16:0/18:3) | 0.15 | -0.03 | 0.33 | 0.0959 | 1 | 0.40515 | 0.1001 |
| PC(16:0/20:0) | -0.10 | -0.28 | 0.08 | 0.262 | 1 | 0.62667 | -0.1157 |
| PC(16:0/20:1) | -0.13 | -0.31 | 0.05 | 0.15 | 1 | 0.48621 | -0.0569 |
| PC(16:0/20:2) | -0.20 | -0.37 | -0.02 | 0.0324 | 1 | 0.24365 | -0.1197 |
| **PC(16:0/20:3)** | **0.41** | **0.25** | **0.58** | **3.28E-06** | 0.00123328 | **0.00123** | 0.2128 |
| PC(16:0/20:4) | 0.06 | -0.12 | 0.24 | 0.516 | 1 | 0.76976 | 0.0050 |
| PC(16:0/20:5) | -0.03 | -0.21 | 0.15 | 0.761 | 1 | 0.91725 | -0.0045 |
| PC(16:0/22:2) | -0.10 | -0.28 | 0.08 | 0.289 | 1 | 0.62667 | -0.0490 |
| PC(16:0/22:3) | 0.18 | 0.00 | 0.36 | 0.0501 | 1 | 0.27924 | 0.1683 |
| PC(16:0/22:4) | 0.19 | 0.01 | 0.37 | 0.0373 | 1 | 0.25972 | 0.1335 |
| **PC(16:0/22:5)** | **0.26** | **0.09** | **0.43** | **0.0043** | 1 | **0.07471** | 0.2388 |
| PC(16:0/22:6) | 0.09 | -0.09 | 0.28 | 0.305 | 1 | 0.62667 | 0.1669 |
| PC(16:1/16:1) | 0.06 | -0.12 | 0.24 | 0.535 | 1 | 0.77369 | 0.0748 |
| **PC(16:1/18:0)** | **0.23** | **0.06** | **0.41** | **0.0104** | 1 | **0.12220** | 0.3098 |
| PC(16:1/18:1) | -0.05 | -0.24 | 0.13 | 0.553 | 1 | 0.79060 | -0.0105 |
| PC(16:1/18:2) | -0.01 | -0.19 | 0.17 | 0.896 | 1 | 0.96042 | 0.0524 |
| **PC(16:1/18:3)** | **0.26** | **0.08** | **0.43** | **0.00484** | 1 | **0.07583** | 0.7827 |
| PC(16:1/20:2) | -0.15 | -0.33 | 0.02 | 0.0938 | 1 | 0.40505 | -0.2561 |
| PC(16:1/20:3) | 0.14 | -0.04 | 0.32 | 0.139 | 1 | 0.47762 | 0.0915 |
| PC(16:1/20:4) | 0.05 | -0.13 | 0.23 | 0.561 | 1 | 0.79740 | -0.0039 |
| PC(18:0/18:0) | 0.18 | 0.01 | 0.36 | 0.0451 | 1 | 0.27351 | 0.0892 |
| PC(18:0/18:1) | 0.19 | 0.02 | 0.37 | 0.0339 | 1 | 0.24585 | 0.1234 |
| PC(18:0/18:2) | 0.00 | -0.18 | 0.18 | 0.972 | 1 | 0.98587 | -0.0034 |
| PC(18:0/18:3) | 0.09 | -0.09 | 0.27 | 0.344 | 1 | 0.65657 | -0.0278 |
| PC(18:0/20:2) | -0.10 | -0.28 | 0.08 | 0.26 | 1 | 0.62667 | -0.0353 |
| **PC(18:0/20:3)** | **0.37** | **0.21** | **0.54** | **2.80E-05** | 0.010528 | **0.00351** | 0.2222 |
| PC(18:0/20:5) | -0.08 | -0.26 | 0.10 | 0.361 | 1 | 0.67717 | -0.0933 |
| PC(18:0/22:4) | -0.10 | -0.28 | 0.08 | 0.302 | 1 | 0.62667 | -0.1490 |
| PC(18:0/22:5) | 0.11 | -0.07 | 0.29 | 0.236 | 1 | 0.58626 | 0.0525 |
| PC(18:1/18:1) | -0.03 | -0.21 | 0.15 | 0.766 | 1 | 0.91725 | 0.0324 |
| PC(18:1/18:2) | 0.00 | -0.18 | 0.18 | 0.97 | 1 | 0.98587 | 0.0062 |
| PC(18:1/18:3) | -0.19 | -0.37 | -0.01 | 0.0402 | 1 | 0.26991 | -0.2236 |
| PC(18:1/20:0) | -0.01 | -0.19 | 0.17 | 0.914 | 1 | 0.96042 | -0.0156 |
| **PC(18:1/20:1)** | **-0.32** | **-0.50** | **-0.15** | **0.000321** | 0.120696 | **0.02012** | -0.2528 |
| PC(18:1/20:2) | -0.15 | -0.33 | 0.03 | 0.1 | 1 | 0.41319 | -0.1732 |
| **PC(18:1/20:3)** | **0.31** | **0.14** | **0.48** | **0.000533** | 0.200408 | **0.02863** | 0.1380 |
| PC(18:1/22:0) | -0.06 | -0.24 | 0.12 | 0.518 | 1 | 0.76976 | -0.0978 |
| PC(18:2/18:2) | -0.19 | -0.36 | -0.01 | 0.0423 | 1 | 0.26998 | -0.1813 |
| PC(18:2/20:0) | -0.12 | -0.30 | 0.06 | 0.193 | 1 | 0.56111 | -0.1135 |
| PC(18:2/20:2) | -0.06 | -0.24 | 0.12 | 0.514 | 1 | 0.76976 | -0.0601 |
| PC(18:2/20:3) | -0.13 | -0.30 | 0.05 | 0.176 | 1 | 0.54243 | -0.2557 |
| **PC(18:2/20:4)** | **0.22** | **0.04** | **0.39** | **0.018** | 1 | **0.16114** | 0.1842 |
| PC(18:2/22:5) | 0.03 | -0.15 | 0.21 | 0.754 | 1 | 0.91725 | -0.0181 |
| PC(20:0/20:3) | 0.01 | -0.17 | 0.19 | 0.917 | 1 | 0.96042 | 0.0467 |
| PE(14:0/22:5) | 0.06 | -0.12 | 0.24 | 0.53 | 1 | 0.77240 | 0.0204 |
| PE(14:1/18:1) | 0.00 | -0.18 | 0.19 | 0.96 | 1 | 0.98354 | -0.0866 |
| PE(16:0/16:0) | -0.10 | -0.28 | 0.08 | 0.274 | 1 | 0.62667 | -0.1640 |
| PE(16:0/16:1) | 0.11 | -0.07 | 0.29 | 0.223 | 1 | 0.58228 | 0.1261 |
| PE(16:0/18:0) | 0.07 | -0.11 | 0.25 | 0.461 | 1 | 0.73138 | 0.0053 |
| PE(16:0/18:1) | 0.00 | -0.18 | 0.18 | 0.978 | 1 | 0.98587 | -0.0924 |
| PE(16:0/18:2) | 0.14 | -0.04 | 0.32 | 0.137 | 1 | 0.47762 | 0.2137 |
| PE(16:0/20:2) | -0.12 | -0.30 | 0.06 | 0.187 | 1 | 0.55660 | -0.0500 |
| PE(16:0/20:3) | -0.01 | -0.19 | 0.17 | 0.915 | 1 | 0.96042 | -0.0337 |
| PE(16:0/20:4) | 0.18 | 0.00 | 0.35 | 0.0552 | 1 | 0.29233 | 0.1380 |
| PE(16:0/20:5) | 0.01 | -0.18 | 0.19 | 0.952 | 1 | 0.98069 | -0.0121 |
| PE(16:0/22:4) | 0.04 | -0.14 | 0.22 | 0.679 | 1 | 0.88647 | -0.0084 |
| PE(16:0/22:5) | 0.14 | -0.04 | 0.32 | 0.134 | 1 | 0.47762 | 0.1091 |
| PE(16:0/22:6) | -0.14 | -0.32 | 0.04 | 0.135 | 1 | 0.47762 | -0.2607 |
| PE(16:1/18:0) | 0.00 | -0.18 | 0.18 | 1 | 1 | 1.00000 | -0.0089 |
| PE(16:1/18:1) | -0.02 | -0.20 | 0.16 | 0.847 | 1 | 0.94055 | -0.1029 |
| PE(16:1/18:2) | -0.13 | -0.31 | 0.05 | 0.155 | 1 | 0.49812 | -0.1895 |
| PE(16:1/20:3) | 0.10 | -0.08 | 0.28 | 0.286 | 1 | 0.62667 | 0.1151 |
| PE(18:0/18:0) | 0.00 | -0.18 | 0.18 | 0.994 | 1 | 0.99665 | -0.0867 |
| PE(18:0/18:1) | -0.14 | -0.32 | 0.04 | 0.129 | 1 | 0.47456 | -0.0747 |
| PE(18:0/18:2) | -0.06 | -0.24 | 0.12 | 0.527 | 1 | 0.77240 | -0.0709 |
| **PE(18:0/18:3)** | **0.31** | **0.14** | **0.48** | **0.000649** | 0.244024 | **0.03050** | 0.3453 |
| PE(18:0/20:2) | -0.12 | -0.30 | 0.06 | 0.199 | 1 | 0.56685 | -0.0868 |
| PE(18:0/20:3) | 0.19 | 0.01 | 0.36 | 0.0438 | 1 | 0.26998 | 0.2461 |
| PE(18:0/20:4) | 0.17 | -0.01 | 0.35 | 0.0653 | 1 | 0.32306 | 0.2538 |
| PE(18:0/20:5) | 0.05 | -0.13 | 0.23 | 0.608 | 1 | 0.83739 | -0.0258 |
| PE(18:0/22:6) | -0.10 | -0.28 | 0.08 | 0.29 | 1 | 0.62667 | -0.1981 |
| PE(18:1/18:1) | 0.03 | -0.15 | 0.22 | 0.714 | 1 | 0.90593 | -0.0111 |
| PE(18:1/18:2) | 0.11 | -0.07 | 0.29 | 0.218 | 1 | 0.57320 | 0.0738 |
| PE(18:1/18:3) | 0.10 | -0.08 | 0.28 | 0.278 | 1 | 0.62667 | 0.0550 |
| PE(18:1/20:3) | 0.09 | -0.09 | 0.27 | 0.306 | 1 | 0.62667 | 0.0494 |
| PE(18:1/20:4) | 0.10 | -0.08 | 0.28 | 0.279 | 1 | 0.62667 | 0.1691 |
| PE(18:1/20:5) | 0.00 | -0.18 | 0.18 | 0.993 | 1 | 0.99665 | -0.0602 |
| PE(18:2/18:2) | 0.16 | -0.02 | 0.34 | 0.0885 | 1 | 0.39148 | 0.1095 |
| PE(18:2/18:3) | -0.06 | -0.24 | 0.12 | 0.489 | 1 | 0.75974 | -0.2366 |
| PE(18:2/20:2) | 0.12 | -0.06 | 0.30 | 0.211 | 1 | 0.57067 | 0.0418 |
| PE(18:2/20:3) | 0.14 | -0.04 | 0.32 | 0.141 | 1 | 0.47762 | 0.1136 |
| PE(18:2/20:4) | -0.02 | -0.20 | 0.16 | 0.822 | 1 | 0.93040 | -0.0433 |
| **PE(18:3/22:5)** | **0.22** | **0.04** | **0.39** | **0.0188** | 1 | **0.16439** | 0.2301 |
| PE(20:0/22:6) | -0.07 | -0.26 | 0.11 | 0.421 | 1 | 0.72121 | -0.2230 |
| PE(20:2/20:4) | -0.03 | -0.21 | 0.15 | 0.764 | 1 | 0.91725 | -0.0932 |
| PE(20:4/20:4) | -0.14 | -0.32 | 0.04 | 0.141 | 1 | 0.47762 | -0.1218 |
| PE(22:3/22:6) | 0.03 | -0.15 | 0.21 | 0.73 | 1 | 0.91725 | 0.0892 |
| PG(14:0/16:0) | -0.12 | -0.30 | 0.06 | 0.21 | 1 | 0.57067 | -0.2412 |
| PG(16:0/16:0) | 0.17 | -0.01 | 0.34 | 0.0728 | 1 | 0.34722 | 0.0132 |
| PG(16:0/18:0) | 0.08 | -0.10 | 0.26 | 0.408 | 1 | 0.72023 | 0.0117 |
| **PG(16:0/18:1)** | **0.26** | **0.09** | **0.44** | **0.00399** | 1 | **0.07471** | 0.1938 |
| **PG(16:0/18:2)** | **0.25** | **0.07** | **0.42** | **0.00703** | 1 | **0.08811** | 0.1516 |
| **PG(16:0/20:2)** | **0.23** | **0.05** | **0.40** | **0.0139** | 1 | **0.14518** | 0.1077 |
| PG(16:0/20:3) | 0.07 | -0.11 | 0.25 | 0.463 | 1 | 0.73146 | 0.0143 |
| PG(18:0/18:0) | 0.09 | -0.09 | 0.27 | 0.339 | 1 | 0.65033 | -0.0142 |
| **PG(18:0/18:1)** | **0.26** | **0.09** | **0.44** | **0.00367** | 1 | **0.07263** | 0.1844 |
| **PG(18:0/18:2)** | **0.29** | **0.12** | **0.47** | **0.00122** | 0.45872 | **0.04165** | 0.2552 |
| PG(18:0/20:4) | 0.16 | -0.01 | 0.34 | 0.0748 | 1 | 0.34722 | 0.0580 |
| PG(18:1/18:1) | 0.18 | 0.00 | 0.36 | 0.0539 | 1 | 0.28952 | 0.0799 |
| **PG(18:1/18:2)** | **0.22** | **0.04** | **0.40** | **0.0158** | 1 | **0.15634** | 0.1317 |
| PI(16:0/16:0) | 0.13 | -0.04 | 0.31 | 0.143 | 1 | 0.48007 | 0.0637 |
| PI(16:0/16:1) | 0.18 | 0.00 | 0.36 | 0.0481 | 1 | 0.27573 | 0.2134 |
| PI(16:0/18:0) | -0.01 | -0.19 | 0.17 | 0.921 | 1 | 0.96193 | 0.0220 |
| PI(16:0/18:1) | 0.08 | -0.10 | 0.26 | 0.413 | 1 | 0.72067 | 0.0878 |
| **PI(16:0/18:2)** | **0.25** | **0.07** | **0.42** | **0.007** | 1 | **0.08811** | 0.2187 |
| PI(16:0/20:3) | 0.02 | -0.16 | 0.20 | 0.82 | 1 | 0.93040 | 0.0079 |
| PI(16:0/20:4) | 0.16 | -0.01 | 0.34 | 0.0739 | 1 | 0.34722 | 0.0888 |
| PI(16:1/18:0) | 0.00 | -0.18 | 0.18 | 0.976 | 1 | 0.98587 | 0.0397 |
| PI(16:1/18:1) | -0.11 | -0.29 | 0.07 | 0.252 | 1 | 0.61527 | -0.1097 |
| PI(18:0/18:1) | -0.06 | -0.24 | 0.12 | 0.545 | 1 | 0.78513 | -0.0866 |
| PI(18:0/18:2) | 0.03 | -0.15 | 0.21 | 0.748 | 1 | 0.91725 | 0.0460 |
| PI(18:0/20:2) | -0.02 | -0.20 | 0.16 | 0.809 | 1 | 0.93040 | -0.0355 |
| **PI(18:0/20:3)** | **0.27** | **0.09** | **0.44** | **0.00347** | 1 | **0.07248** | 0.2013 |
| PI(18:0/20:4) | 0.10 | -0.08 | 0.28 | 0.267 | 1 | 0.62667 | 0.1169 |
| PI(18:0/20:5) | -0.09 | -0.27 | 0.09 | 0.313 | 1 | 0.62935 | -0.0391 |
| PI(18:0/22:4) | -0.03 | -0.21 | 0.15 | 0.741 | 1 | 0.91725 | -0.0553 |
| PI(18:1/18:1) | -0.12 | -0.30 | 0.06 | 0.212 | 1 | 0.57067 | -0.0802 |
| PI(18:1/18:2) | 0.03 | -0.15 | 0.22 | 0.706 | 1 | 0.90419 | -0.0128 |
| PI(18:1/20:3) | -0.02 | -0.20 | 0.16 | 0.839 | 1 | 0.93721 | -0.0126 |
| PI(18:2/18:2) | -0.01 | -0.19 | 0.17 | 0.899 | 1 | 0.96042 | -0.1415 |
| PS(16:0/18:0) | -0.07 | -0.26 | 0.11 | 0.419 | 1 | 0.72121 | -0.0456 |
| **PS(16:0/18:1)** | **-0.22** | **-0.39** | **-0.04** | **0.0177** | 1 | **0.16114** | -0.1578 |
| PS(18:0/18:0) | 0.17 | -0.01 | 0.35 | 0.0605 | 1 | 0.31147 | 0.2625 |
| **PS(18:0/18:1)** | **-0.22** | **-0.40** | **-0.04** | **0.0158** | 1 | **0.15634** | -0.2830 |

**Supplementary Table 6.** Interaction of 6 lipids and sex using multiple linear regression.

| **lipid×sex** | **Estimate** | **CI_lower** | **CI_upper** | **Pr(>\|t\|)** | **bonferroni** | **BH** |
| --- | --- | --- | --- | --- | --- | --- |
| PC(O-16:0/18:3) | 0.05 | -0.28 | 0.38 | 0.79 | 1 | 0.7896 |
| PC(P-16:0/18:2) | -0.11 | -0.44 | 0.23 | 0.59275 | 1 | 0.7896 |
| PC(16:0/20:3) | -0.09 | -0.42 | 0.24 | 0.6411 | 1 | 0.7896 |
| **PC(16:1/18:3)** | **0.44** | **0.11** | **0.76** | **0.0276** | **0.1656** | **0.1656** |
| PC(18:1/20:1) | 0.14 | -0.16 | 0.45 | 0.44217 | 1 | 0.7896 |
| PE(18:0/18:3) | 0.08 | -0.25 | 0.41 | 0.6807 | 1 | 0.7896 |

PC: phosphatidylcholine

PC(O): alkyl-PC

PC(P): alkenyl-PC

PE: phosphatidylethanolamine

**Supplementary Table 7.** Odds Ratios (Ors) and Significance of three PC metabolites and the covariables in Multivariable Logistic Regression Models.

|  | Model 1 |  | Model 1b |  | Model 2 |  | Model 3 |  | Model 4 |  |
| --- | --- | --- | --- | --- | --- | --- | --- | --- | --- | --- |
| Variable | OR (95% CI) | P_fdr | OR (95% CI) | P_fdr | OR (95% CI) | P_fdr | OR (95% CI) | P_fdr | OR (95% CI) | P_fdr |
| PC(P-16:0/18:2) | 2.41  (1.42-4.52) | 0.0156 |  |  | 2.61  (1.46-5.31) | 0.031 | 2.69  (1.49-.58) | 0.036 | 6.08  (2.65-17.74) | 0.0014 |
| PC(16:0/20:3) | 2.43  (1.44-4.48) | 0.0114 |  |  | 3.23  (1.76-6.63) | 0.005 | 3.24  (1.74-.81) | 0.0072 | 3.59  (1.83-8.20) | 0.0049 |
| PC(16:1/18:3) | 2.91  (1.66-5.71) | 0.0036 |  |  | 3.74  (2.01-7.85) | 0.001 | 3.83  (2.00-.42) | 0.0024 | 7.79  (3.15-4.97) | 7.00E-04 |
| z_age |  |  | 1.30  (0.88-1.97) | 0.7776 | 0.914  (0.52-1.59) | 1 | 0.90  (0.50-.59) | 1 | 0.81  (0.42-1.54) | 1 |
| sexmale |  |  | 1.19  (0.54-.61) | 1 | 0.263  (0.07-0.82) | 0.287 | 0.25  (0.06-0.80) | 0.33 | 0.06  (0.01-0.30) | 0.0126 |
| zBMI |  |  |  |  |  |  | 1.72  (1.00-3.26) | 0.822 | 2.10  (1.06-4.79) | 0.371 |
| MD_score |  |  |  |  |  |  |  |  | 3.17  (1.81-6.35) | 0.0021 |

PC: phosphatidylcholine

PC(P): alkenyl-PC

z_age: age z-score

zBMI: BMI z-score

MD_score: metabolic disorder score from PC1 in PCA analysis of TG, TC, HDL-C, LDL-C, and uric acid.

**Supplementary Table 8.** Sensitivity analysis of lipid associated using P75 FPG cutoff

|  | **OR** | **CI_lower** | **CI_upper** | **p** |
| --- | --- | --- | --- | --- |
| PC(P-16:0/18:2) | 2.01 | 1.20 | 3.58 | 0.0112 |
| PC(16:0/20:3) | 2.12 | 1.21 | 4.02 | 0.0128 |
| PC(16:0/20:3) | 1.62 | 0.95 | 2.84 | 0.0791 |

*Model adjusted for sex and BMI. Hyperglycemia defined as FPG ≥ P75 (5.71 mmol/L). Cases: n = 27.

PC: phosphatidylcholine

PC(P): alkenyl-PC
